# High-throughput and Sensitive Immunopeptidomics Platform Reveals Profound Interferonγ-Mediated Remodeling of the Human Leukocyte Antigen (HLA) Ligandome*[Fn FN2]

**DOI:** 10.1074/mcp.TIR117.000383

**Published:** 2017-12-14

**Authors:** Chloe Chong, Fabio Marino, HuiSong Pak, Julien Racle, Roy T. Daniel, Markus Müller, David Gfeller, George Coukos, Michal Bassani-Sternberg

**Affiliations:** From the ‡Ludwig Institute for Cancer Research, University of Lausanne, 1066 Epalinges, Switzerland;; §Department of Oncology, University Hospital of Lausanne, 1011 Lausanne, Switzerland;; ¶Service of Neurosurgery, University Hospital of Lausanne, 1011 Lausanne, Switzerland;; ‖Vital IT, Swiss Institute of Bioinformatics, 1015 Lausanne, Switzerland;; **Swiss Institute of Bioinformatics, 1015 Lausanne, Switzerland

## Abstract

Comprehensive knowledge of the human leukocyte antigen (HLA) class-I and class-II peptides presented to T-cells is crucial for designing innovative therapeutics against cancer and other diseases. However methodologies for their purification for mass-spectrometry analysis have been a major limitation. We designed a novel high-throughput, reproducible and sensitive method for sequential immuno-affinity purification of HLA-I and -II peptides from up to 96 samples in a plate format, suitable for both cell lines and tissues. Our methodology drastically reduces sample-handling and can be completed within five hours. We challenged our methodology by extracting HLA peptides from multiple replicates of tissues (*n* = 7) and cell lines (*n* = 21, 10^8^ cells per replicate), which resulted in unprecedented depth, sensitivity and high reproducibility (Pearson correlations up to 0.98 and 0.97 for HLA-I and HLA-II). Because of the method's achieved sensitivity, even single measurements of peptides purified from 10^7^ B-cells resulted in the identification of more than 1700 HLA-I and 2200 HLA-II peptides. We demonstrate the feasibility of performing drug-screening by using ovarian cancer cells treated with interferon gamma (IFNγ). Our analysis revealed an augmented presentation of chymotryptic-like and longer ligands associated with IFNγ induced changes of the antigen processing and presentation machinery. This straightforward method is applicable for basic and clinical applications.

The rich repertoire of peptides presented by HLA class I (HLA-I)^1^ and HLA class II (HLA-II) complexes, referred to as the immunopeptidome, reflects the health state of a cell. HLA-bound peptides (HLAp) derived from cancer-specific and mutated proteins, pathogens and self-peptides in case of autoimmunity, serve as leading targets for T-cell recognition. In recent years the remarkable clinical efficacy of immune checkpoint blockade therapies has motivated researchers to discover immunogenic T-cell epitopes that mediate disease control (1) or improved survival for development of personalized vaccines (2–5).

Presently, mass spectrometry (MS) is the only unbiased methodology to comprehensively interrogate the *in vivo* naturally presented HLAp repertoire (6), in human cell lines (7–9), tumor tissues (10–12) and body fluids such as plasma (13). Importantly, pioneering proof-of-concept studies have shown that this technology has matured to the extent that identification of clinically relevant mutated antigens in humans has become reality (14–17). In the field of immunology, this methodology is perceived as highly promising although not ready yet for its implementation in clinical settings because of its low sensitivity and robustness (2).

HLA-I and HLA-II complexes have key roles in modulation of immune responses and are distinguishable by the type of cells that express and recognize them and by the distinct biogenesis of the presented peptides (18). The repertoire of the presented immunopeptidome is constantly modulated by source protein expression levels, post translational modifications, and by several enzymes, chaperones and transporters that comprise the cellular antigen processing and presentation machinery (APPM). Cellular perturbation could affect this machinery at multiple levels, leading to the presentation of an altered peptidome. So far, the assessment of differential immunopeptidomics has been mainly unexplored because of technical limitations related to low throughput and reproducibility of existing methodologies (19, 20).

Immunopeptidomics is based on immunoaffinity purification (IP) of HLA complexes from mild detergent solubilized lysates, followed by extraction of the HLAp. The extracted peptides are then separated by chromatography and directly injected into a mass spectrometer. With the new generation of mass spectrometer instrumentations, thousands of HLAp can be readily identified per sample (7, 21).

The most critical step in the immunopeptidomics pipeline is the sample preparation as it determines the overall peptide yield and reproducibility. The entire workflow is laborious, typically spanning over 3 to 5 days, and is often limited to a few samples at a time (22). The above-mentioned bottlenecks pose severe restrictions on implementing this methodology for robust clinical applications and for LFQ comparative studies such as antigen presentation on infection (23), drug treatments or association of particular HLA alleles with autoimmunity (24).

In this work we set out to develop the first high-throughput method for IP of HLAp for MS-based immunopeptidomics, suitable for both basic and translational studies, where thousands of unique HLA-Ip and -IIp can be readily identified in a single IP procedure from cell lines and tissue samples. As IP of clinically relevant samples are often hindered by scarcely available amounts (12, 25–27), we decided to challenge the sensitivity of our platform by immunopurifying HLAp from as low as 10^7^ B-cells. Furthermore, we also demonstrated the feasibility of performing comparative screening using an ovarian cancer cell line treated with the pro-inflammatory cytokine IFNγ. IFNγ is a well-known master regulator of immune modulation that up-regulates antigen presentation on target cells (28). Here, for the first time, we captured IFNγ-mediated modulation of specific components of the APPM which resulted in qualitative and quantitative alterations of the presented HLAp repertoire. Specifically, we discovered an enhanced presentation of chymotryptic-like ligands, as well as longer ligands deriving from nested sets on IFNγ treatment.

## EXPERIMENTAL PROCEDURES

### 

#### 

##### Cell Lines

EBV-transformed human B-cell lines JY (ATCC® 77442™, Manassas, Virginia), CD165, PD42, CM467, RA957 (a gift from Pedro Romero, Ludwig Cancer Research Lausanne) were maintained in RPMI 1640 + GlutaMAX medium (Life Technologies, Carlsbad, CA) supplemented with 10% heat-inactivated fetal bovine serum (FBS) (Dominique Dutscher, Brumath, France) and 1% Penicillin/Streptomycin Solution (BioConcept, San Diego, CA). UWB.1 289 ovarian carcinoma cells (ATCC® CRL-2945™) were maintained in a 1:1 mix of HuMEC Ready medium (Thermo Fisher Scientific, Waltham, MA) supplemented with HuMEC Supplement Kit (Thermo Fisher Scientific) and RPMI 1640 + GlutaMAX medium, with addition of 1% Penicillin/Streptomycin Solution and 3% heat-inactivated FBS.

Cells were grown to the required cell amount, collected by centrifugation at 1200 rpm for 5 min, washed twice with ice cold PBS and stored as dry cell pellets at −20 °C until use. For the *in vitro* treatment of UWB. 1 289 cells with human IFNγ (Miltenyl Biotec, Bergisch Gladbach, Germany), cells were grown to 1.5 × 10^8^ in quadruplicates both for control and treatment. For treatment, cells were exposed to 100 IU/ml IFNγ for 24 h, detached with Accutase (Thermo Fisher Scientific), counted and washed twice with cold PBS before storage at −20 °C.

All cells were tested negative for mycoplasma contamination. High resolution 4-digit HLA-I and HLA-II typing was performed for all cell lines at the Laboratory of Diagnostics, Service of Immunology and Allergy, CHUV, Lausanne and provided in supplemental Table S1.

##### Patient Material

T-cells were expanded from two melanoma tumors as previously described (29) following established protocols (30, 31). Briefly, fresh tumor samples were cut in small fragments and placed in 24-well plate containing RPMI CTS grade (Life Technologies), 10% Human serum (Valley Biomedical, Winchester, VA), 0.025 m HEPES (Life Technologies), 55 μmol/l 2-Mercaptoethanol (Life Technologies) and supplemented with a high concentration of IL-2 (Proleukin, 6,000 IU/ml, Novartis, Basel, Switzerland) for 3 to 5 weeks. Following this initial pre-rapid expansion, tumor infiltrating lymphocytes (TILs) were then expanded in using a rapid expansion protocol approach. To do so, 25 × 10^6^ TILs were stimulated with irradiated feeder cells, anti-CD3 (OKT3, 30 ng/ml, Miltenyl biotec) and high dose IL-2 (3,000 IU/ml) for 14 days. The final cell product was washed and prepared using a cell harvester (LoVo, Fresenius Kabi, Lake County, IL). On receival of TIL samples, the cells were washed with PBS on ice, aliquoted to a cell count of 1 × 10^8^ and stored as dry pellets at −80 °C until use.

Snap frozen meningioma tissues from patients (3830-NJF, 3849-BR, 3912-BAM, 3865-DM) were obtained from the University Hospital of Lausanne (CHUV, Lausanne, Switzerland).

Informed consent of the participants was obtained following requirements of the institutional review board (Ethics Commission, CHUV). Protocol F-25/99 has been approved by the local Ethics committee and the biobank of the Lab of Brain Tumor Biology and Genetics.

##### Generation of Antibody-crosslinked Beads

W6/32 and HB145 monoclonal antibodies were purified from the supernatant of HB95 (ATCC® HB-95™) and HB145 cells (ATCC® HB-145™) grown in CELLLine CL-1000 flasks (Sigma-Aldrich, St. Louis, MI) using protein-A Sepharose 4B (Pro-A) beads (Invitrogen, Carlsbad, CA). Antibodies were cross-linked to Pro-A beads at a concentration of 5 mg of antibodies per 1 ml volume of beads. For this purpose, the antibodies were incubated with the Pro-A beads for 1 h at room temperature. Chemical cross-linking was performed by addition of Dimethyl pimelimidate dihydrochloride (Sigma-Aldrich) in 0.2 m Sodium Borate buffer pH 9 (Sigma-Aldrich) at a final concentration of 20 mm for 30 min. The reaction was quenched by incubation with 0.2 m ethanolamine pH 8 (Sigma-Aldrich) for 2 h. Cross-linked antibodies were kept at 4°C until use.

##### High-throughput Purification of HLA Class-I and -II Complexes

**Fig. 1.**
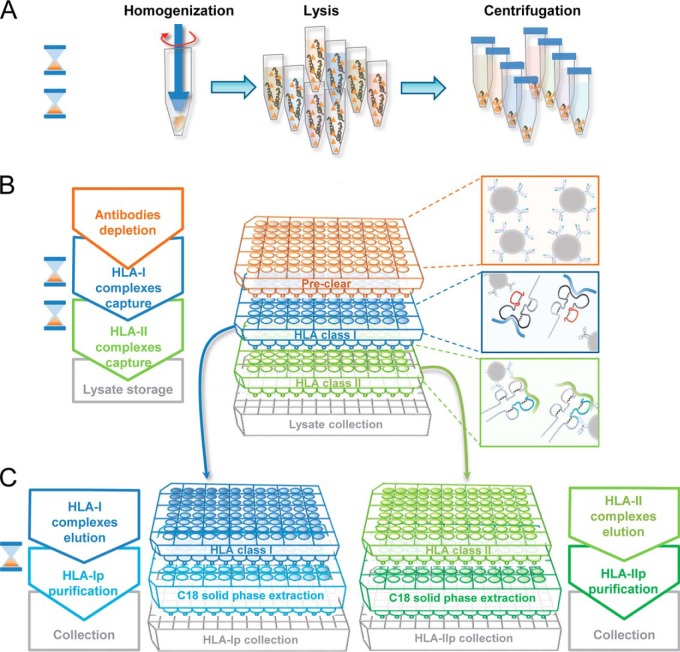
**Outline of the high-throughput immunopurification workflow using a plate format.** *A*, Tissues are first homogenized, lysed with mild detergents and cleared with a centrifugation step. *B*, To enable sequential loading of the lysates on multiple affinity resins, cleared lysates are loaded on stacked plates containing firstly, Pro-A beads for depletion of tissue endogenous antibodies, then anti-HLA class I and II antibodies cross-linked to Pro-A beads for direct enrichment of HLA class I and II complexes. *C*, Affinity plates containing the captured HLA complexes are separated, washed individually and stacked on C18 plates. HLA class I and II complexes are then eluted on the C18 plates. Peptide and protein fractions are then recovered separately. Each step is timed with the hourglass symbol that is equivalent to about one hour.

For high-throughput HLA-I and -II purification, we employed the Waters Positive Pressure-96 Processor (Waters, Milford, MA). For IPs, we used the 96-well single-use micro-plate with 3 μm glass fiber and 10 μm polypropylene membranes which are compatible with the processor and are commercially available (ref number: 360063, Seahorse Bioscience, North Billerica, MA). The positive pressure processor was used in each step of the procedure to generate homogenous flow of liquid through the plates. The suggested applied pressure is in the range of 3–5 psi. The following procedure is also exemplified in Fig. 1.

Preparation of lysates: In the Plate 1 experiment (see supplemental Table S2) we purified the HLA-I and II peptidome from JY, CD165, PD42, CM467, RA957, TIL1, and TIL3. Cell lysis was performed with PBS containing 0.25% sodium deoxycholate (Sigma-Aldrich), 0.2 mm iodoacetamide (IAA) (Sigma-Aldrich), 1 mm EDTA, 1:200 Protease Inhibitors Mixture (Sigma-Aldrich), 1 mm Phenylmethylsulfonylfluoride (Roche, Basel, Switzerland), 1% octyl-beta-d glucopyranoside (Sigma-Alrich) at 4 °C for 1 h. In general, lysis buffer was added to the cells at a concentration of 1 × 10^8^ cells/ml. Lysates were cleared by centrifugation with a table-top centrifuge (Eppendorf Centrifuge, Hamburg, Germany) at 4 °C at 14,200 rpm for 50 min. For each cell line, lysate from a total of 3 × 10^8^ cells were pooled and evenly distributed as 1 × 10^8^ triplicates into designated wells. Mock wells were incorporated into the experimental set-up, whereby wells contained anti-HLA-I and HLA-II cross-linked beads without addition of lysate. In the Plate 2 experiment **(**supplemental Table S2), snap-frozen meningioma tissue samples were placed in tubes containing ice cold lysis buffer (mentioned above) and homogenized on ice in 3–5 short intervals of 5 s each using an Ultra Turrax homogenizer (IKA, T10 standard, Staufen, Germany) at maximum speed. For one gram of tissue, 10 ml of lysis buffer was required. Lysates were cleared by centrifugation at 25,000 rpm in a high-speed centrifuge (Beckman Coulter, JSS15314, Nyon, Switzerland) at 4 °C for 50 min. To test the sensitivity of our method (Plate 3, see supplemental Table S2), we extracted HLA-I and -II peptides from 10, 30, 50, and 70 million cells as described above and we split the lysate of 1.6 × 10^8^ CD165 B-cells proportionally to the desired cell amount; this was performed in triplicates. Lastly, four biological replicates of UWB.1 289 cells untreated and treated with IFNγ (1.5 × 10^8^ cells each replicate) were processed in parallel for HLAp purification (Plate 4, see supplemental Table S2).

Preparation of plates: First, empty plates' wells were washed and equilibrated with 1 ml of 100% ACN (Sigma-Aldrich), followed by 1 ml of 0.1% TFA (Merck Millipore, Billerica, Massachusetts) and lastly with 2 ml of 0.1 m Tris-hydrochloric acid (HCl) pH 8 (Thermo Fisher Scientific). Anti-pan HLA-I and HLA-II antibodies cross-linked to beads were loaded on their respective plates (named “HLA class I” and “HLA class II,” see Fig. 1) at a final bead volume of 75 μl in 0.1 m Tris-HCl. For tissue samples, a depletion step of endogenous antibodies was required. Therefore, an additional plate (named “Pre-clear” plate) with wells containing 100 μl Pro-A beads was prepared. The beads alone or antibodies cross-linked to beads were conditioned with lysis buffer before lysate loading.

Affinity purification of HLA complexes using the processor: As represented in Fig. 1, for tissue purification, three plates were sequentially stacked together; the Pre-clear on top, followed by the HLA class I, HLA class II and lastly, collection or waste plates. In this manner, we sequentially depleted the endogenous antibodies and immuno-affinity purified HLA class I and II complexes without intermediate steps. For cell line preparation, the pre-clear plate is not necessary. The lysates were loaded on the first plate and flowed by gravity through the preclear (for tissues only), HLA class I and II plates at 4 °C. HLA class I and II plates were then washed separately (Fig. 1) using the processor as follows: 4 times 2 ml of 150 mm sodium chloride (NaCl) (Carlo-Erba, Val de Reuil, France) in 20 mm Tris-HCl pH 8, 4 times 2 ml of 400 mm NaCl in 20 mm Tris-HCl pH 8 and again with 4 times 2 ml of 150 mm NaCl in 20 mm Tris-HCl pH 8. Finally, we washed the beads twice with 2 ml of 20 mm Tris-HCl pH 8.

Purification of HLA-I and HLA-II peptides: Two Sep-Pak tC18 100 mg Sorbent 96-well plates (named “C18 solid phase extraction” plate) (ref number: 186002321, Waters) were required for the purification and concentration of HLA-I and HLA-II peptides. Each C18 plate was handled separately. Firstly, we conditioned the plates with 1 ml of 80% ACN in 0.1% trifluoroacetic acid (TFA) and then with 2 ml of 0.1% TFA. The affinity plate was stacked on top of the C18 plate to achieve direct elution of the HLA complexes and the bound peptides with 500 μl 1% TFA. The use of TFA leads to complete denaturation of antibodies and results in a high recovery of HLAp. This is followed by washing the C18 wells with 2 ml of 0.1% TFA. Thereafter, we eluted the HLA-I peptides with 500 μl of 28% ACN in 0.1% TFA. HLA-II peptides were eluted from the class II C18 plate with 500 μl of 32% ACN in 0.1% TFA. Both HLA-I and -II peptides elutions were transferred into eppendorf tubes. Recovered HLA-I and -II peptides were dried using vacuum centrifugation (Concentrator plus Eppendorf) and stored at −20 °C. The overall time required for sample drying may vary according to the specification of the vacuum centrifuge, the user settings and amount of samples.

HLA class I and II heavy chains and the β2m molecules were recovered from the C18 plates using 300 μl of 80% ACN in 0.1% TFA. The samples were dried down and re-suspended in 30 μl 0.1% TFA. One-third of each fraction was loaded onto an SDS-gel for visual inspection of HLA complexes by SDS-electrophoresis.

##### Sample Preparation for Proteomics Analysis

The four biological replicates of IFNγ treated and untreated UWB.1 289 cells were re-suspended in lysis buffer composed of 8 m Urea (Biochemica, Billingham, UK) and 50 mm ammonium bicarbonate (AMBIC, Sigma-Aldrich) pH 8. The cell lysates were sonicated in the Bioruptor instrument (Diagenode, B01020001, Seraing, Belgium) for 15 cycles, maximum mA for 30 s each cycle. Subsequently, centrifugation at 20,000 × *g* at 4 °C for 30 min separated the soluble from the insoluble protein fractions. The soluble fraction was collected and the protein concentration of the lysates was determined by a Bradford protein assay. Proteins were reduced with a final concentration of 5 mm DTT (Sigma-Aldrich) at 37 °C for 60 min, followed by alkylation with a final concentration of 15 mm iodoacetamide (IAA, Sigma-Aldrich) at room temperature for 60 min in the dark. After the alkylation step the digestion was carried out with a mixture of endoproteinase Lys-C and Trypsin (Trypsin/Lys-c Mix, Promega, Madison, WI). The first step consists of endoproteinase Lys-C digestion for 4 h at 37 °C with a protein to enzyme ratio of 50:1 (w/w). Subsequently, the samples were diluted 8 times with 50 mm AMBIC to a Urea concentration of 1 m. The second step of digestion was performed with Trypsin overnight at 37 °C with a substrate to enzyme ratio of 50:1 (w/w). After digestion, the samples were acidified with formic acid (FA) and desalted on C18 spin columns (Harvard Apparatus, Holliston, MA). Samples were further fractionated using 2 layers of strong-cation-exchange (SCX) discs (Empore, Sigma-Aldrich) inserted into 20 μl StageTips generated in-house. Centrifugation was performed at up to 500 rcf on a tabletop centrifuge. Three fractions were collected by eluting with 75 mm ammonium acetate (NH_4_AcO) pH 4 (Sigma-Aldrich), 200 mm NH_4_AcO pH 5 and 5% Ammonia (Merck, Corsier-sur-Vevey, Switzerland) in 80% ACN pH 12. The fractions were dried and resuspended in 0.1% TFA for desalting on C18 spin columns. Finally, the samples were dried and resuspended in 2% ACN in 0.1% FA (Thermo Fisher Scientific).

##### LC-MS/MS Analysis

Before MS analysis HLA-I and HLA-II peptide samples were re-suspended in 9 μl of 0.1% FA and 1/3 or ½ of the sample volume were placed in the UHPLC autosampler (as indicated in supplemental Table S2), whereas half of each of the SCX fractions were taken. For HLA-Ip, we used the following gradient with a flow rate of 250 nl/min using a mix of 0.1% FA (buffer A) and 0.1% FA in 80% ACN (buffer B): 0–5 min (5% B); 5–85 min (5–35% B); 85–100 min (35–60% B); 100–105 min (60–95% B); 105–110 min (95% B); 110–115 min (95–2% B) and 115–125 min (2% B). For HLA-II peptidomics, the gradient consisted of: 0–5 min (2–5% B); 5–65 min (5–30% B); 65–70 min (30–60% B); 70–75 min (60–95% B); 75–80 min (95% B), 80–85 min (95–2% B) and 85–90 min (2% B). Proportionally shorter gradients of 1 h were used for the third experimental set-up (Plate 3) where the HLA-p were extracted from 10, 30, and 50 million cells. For proteomics, the gradient was as such: 0–5 min (2–5% B); 5–30 min (5–9% B); 30–180 min (9–22% B); 180–230 min (22–35% B); 230–250 min (35–60% B); 250–255 min (60–95% B); 255–260 min (95% B); 260–265 min (95–5% B) and 265–270 min (5% B).

All samples were acquired using the nanoflow UHPLC Easy nLC 1200 (Thermo Fisher Scientific, LC140) coupled online to a QExactive HF Orbitrap mass spectrometer (Thermo Fischer Scientific) with a nanoelectrospray ion source (Sonation, PRSO-V1, Baden-Württemberg, Germany). We packed the uncoated PicoTip 8 μm tip opening with 75 μm i.d. × 50 cm long analytical columns with ReproSil-Pur C18 (1.9 μm particles, 120 Å pore size, Dr. Maisch GmbH, Ammerbuch, Germany). Mounted analytical columns were kept at 50 °C using a column oven.

For HLAp, data was acquired with data-dependent “top10” method, which isolates within a 1.2 *m*/*z* window the ten most abundant precursor ions and fragments them by higher-energy collision dissociation (HCD) at normalized collision energy of 27%. For proteomics, data-dependent “top15” method was used. The mass spectrometer scan range was set to 300 to 1650 *m*/*z* with a resolution of 60,000 (200 *m*/*z*) and an AGC target value of 3e6 ions for HLAp, whereas for proteomics, the mass spectrometer scan range was set to 300 to 800 *m*/*z.* For MS/MS, AGC target values of 1e5 were used with a maximum injection time of 120 ms (HLAp) or 25 ms (proteomics) at set resolution of 15,000 (200 *m*/*z*). For HLA-I peptidomics, in case of assigned precursor ion charge states of four and above, no fragmentation was performed. For HLA-II peptidomics, in case of assigned precursor ion charge states of one, and from six and above, no fragmentation was performed. The peptide match option was disabled. For proteomics, in case of unassigned precursor ion charge states or a charge state of one, no fragmentation was performed and the peptide match option was set to “preferred.” The dynamic exclusion of precursor ions from further selection was set for 20 s.

##### Database Search

We employed the MaxQuant computational proteomics platform version 1.5.5.1 (32) to search the peak lists against the UniProt databases (Human 42,148 entries, March 2017) and a file containing 247 frequently observed contaminants. N-terminal acetylation (42.010565 Da) and methionine oxidation (15.994915 Da) were set as variable modifications. As the IP lysis buffer contains IAA we included in an additional search also cysteine carbamidomethylation (57.021463 Da) as a variable modification. For proteomics, a fixed modification of cysteine carbamidomethylation (57.021463 Da) was used. The second peptide identification option in Andromeda was enabled. A false discovery rate (FDR) of 0.01 and no protein FDR was set for peptidomics analysis whereas a protein FDR of 0.01 was set for proteomic analysis. The enzyme specificity was set as unspecific for peptidomics analysis, whereas C-terminal specificity for K and R, and max 2 miscleavages were chosen for analysis of proteomics samples. Possible sequence matches were restricted to 8 to 25 amino acids (a.a.), a maximum peptides mass of 4600 Da. The initial allowed mass deviation of the precursor ion was set to 6 ppm and the maximum fragment mass deviation was set to 20 ppm. Where indicated, we enabled the “match between runs” option, which allows matching of identifications across different replicates of the same biological sample in a time window of 0.5 min and an initial alignment time window of 20 min. For proteomic analysis, “match between runs” module was enabled between all samples and label-free quantification (LFQ) was enabled in the MaxQuant environment (33).

##### Experimental Design and Statistical Rationale

A detailed description of the immunopeptidomic experimental design, including naming of samples and their positions on the plates, RAW MS file names, and assignment of biological and technical replicates are provided in supplemental Table S2. We used the Perseus computational platform version 1.5.5.3 (34) for all statistical analysis, unless otherwise indicated. For immunopeptidomics, we used the “peptides” MaxQuant output table. Peptides matching to reverse and contaminants were filtered out. The values of peptide intensities were log2 transformed and Pearson correlations of the intensities were calculated for each experiment. For Plate 1 and 2 experiments, “match between runs” was enabled only between same biological samples and separately for HLA class I and II peptides. For Plate 3 experiment, “match between runs” was enabled only between the replicates of similar lysate dilution (*i.e.* all the 3 replicates corresponding to 10 million cells) and separately for HLA class I and II peptides. For the bioinformatics analysis of the IFNγ (Plate 4) experiment, the intensities were normalized using “width normalization” option in Perseus. Briefly, for each sample, the first, second and third quartiles (q1, q2, q3) are calculated from the distribution of all values. The median (q2) is subtracted from each value to center the distribution. Then we divide by the width in an asymmetric way. All values that are positive after subtraction of the median are divided by q3 - q2 whereas all negative values are divided by q2 - q1. Missing intensity values were imputed by drawing random numbers from a Gaussian distribution with a standard deviation of 20% in comparison to the standard deviation of measured peptide abundances. Volcano plots of modulations in the relative intensities of HLA ligands on IFNγ treatment were created. Each dot represents a unique HLA-I peptide. Log2-fold changes of their abundance are indicated on the *x* axis and the corresponding significance levels were calculated by two-sided unpaired *t* test with a FDR of 0.01 and S0 of 1. For proteomic analysis of UWB.1 289 cell line treated with IFNγ, LFQ intensities of proteins were retrieved from the “ProteinGroups” MaxQuant output table, were log2 transformed and a filter was set for at least 3 valid values in either the control or IFNγ treated groups. Missing intensities were imputed as described above and a volcano plot was generated where log2-fold changes of IFNγ *versus* control group are indicated on the *x* axis and the corresponding significance levels were calculated by two-sided unpaired *t* test with a FDR of 0.01 and S0 of 0.2.

For the analysis of tryptic- and chymotryptic-like ligands in the immunopeptidome, we grouped the peptides based on their C-terminal specificities: K and R a.a. for tryptic-like ligands and A, F, I, L, M, V, and Y for chymotryptic-like ligands. Affinities to the corresponding allotypes expressed in the UWB.1 289 cell line were predicted for all 8 to 15 mer eluted peptides identified using NetMHC4.0 (35). Binding predictions were assigned to peptides only if they were predicted to bind to only one HLA allotype. The threshold for binding was set to rank <2% and the respective affinity values in nm were extracted. Sequence motifs were calculated and visualized from Gibbscluster-2.0e (36) and Seq2logo (37). For length distribution, affinity and hydrophobicity analyses, we enabled the option of “match between runs” only within the control and IFNγ groups and used the uniquely identified peptides in control and IFNγ treatment samples for comparison. The same list of peptides was used for comparing predicted binding affinities between control and IFNγ treated samples. Hydrophobicity scores were calculated online with https://www.protpi.ch/Calculator. Their significance levels of control and IFNγ treated samples were calculated using a two-sided unpaired *t* test. IceLogo was used to calculate the statistics to find over-represented a.a. in each position of HLA-B*07:02, -A*68:01 and -A*03:01 predicted binders of the IFNγ dataset compared with the control, with a *p* value cut-off of 0.01(38). The normalized output tables were parsed using a Java program to retain proteins with matched overlapping HLA-Ip sequences. We determined nested pairs of peptides containing the same core region (referred as “short”) differing by up to five a.a. to the left (N-terminal) or to the right (C-terminal) (referred as “long”). Intensity changes on IFNγ treatment were calculated as Normalized log2-intensity difference = log2((IFNγ_long_-ctrl_long_)/(IFNγ_short_-ctrl_short_)). For in-depth analysis of C-terminal extensions, we paired short and long peptide versions based on whether they remained tryptic-like, chymo tryptic-like, or if their cleavage specificities were switched. The *p* values were calculated using a one-sided *t* test, where the null hypothesis represented zero change. Statistical calculations and plots were performed in R (www.r-project.org).

##### Synthetic Peptides

15 peptides (PEPotech Heavy grade 3, Thermo Fisher Scientific) with Alanine and Leucine C-terminal were selected based on their high intensities and retention time distribution from previously measured CD165 HLA-I samples. We mixed all the heavy-labeled peptides (listed in supplemental Table S3) together and desalted them on a C18 spin column. Peptides were dried to obtain 10,000 pmol of each peptide in the mixture. To test the level of cross-contamination between wells as well as reproducibility, we spiked-in the 15 peptides at 50 pmol immediately after the peptides were placed onto the C18 plate for the three replicates of CD165 HLA-I samples.

To measure the total abundance of synthetic peptides, the area under the curve (AUC) of extracted ion chromatograms for charge states z = 1+, z = 2+, and z = 3+ were calculated and summed to obtain the total signal of a given peptide. The log2 ratio between heavy and light peptides was then calculated and the mean, standard deviation and coefficient of variation (CV) were assigned for 3 exemplary synthetic peptides (see supplemental Table S4) as an example of reproducibility between the three replicates.

##### Gibbs Clustering Analysis for HLA-II Peptides

Gibbscluster-2.0e (31) was run independently for each sample using all HLA-IIp identified in a given sample, with the default options except that the number of clusters was tested between 1 and 6, the number of seeds for initial conditions was set to 5, the initial Monte Carlo temperature was 1.5, and we enabled the preference for hydrophobic a.a. at P1. The number of motifs plotted for each sample in supplemental Fig. S2 corresponds to the best number of motifs as determined by GibbsCluster.

We determined the reference binding motifs for each HLA-II allele based on peptides annotated in the immune epitope database (IEDB) as positive, positive-high, positive-intermediate and positive-low (39). Here Gibbscluster-2.0e was run separately per allele, with the same parameters mentioned above yet by considering a single cluster. Sequence logos were drawn with Seq2logo, based on Shannon entropy, without any sequence weighting nor Blosum correction (37).

##### FACS Analysis of HLA-I and -II Expression

To analyze cell surface expression of HLA-I and -II of UWB.1 289 cells on IFNγ treatment for 24 h, cells were stained with anti-HLA-A,B,C PerCP/Cy5.5 and anti-HLA-DR DP DQ FITC, or isotype-matched controls (Biolegend, San Diego, CA). Dead cells were measured using DAPI staining (PanReac Applichem, Darmstadt, Germany). Data was acquired using a LSR II SORP instrument (Beckton Dickinsons) and analyzed with the FlowJo Software version 10.3.

## RESULTS

### 

#### 

##### Development of a High-throughput and In-depth Immunopeptidomics Method

In an attempt to improve the sample preparation for MS-based immunopeptidomics, we revisited several recently published studies (7, 12, 17, 25–27, 40–50). Although most reported methods are similar and based on common IP procedures, our literature study systematically revealed insufficient description of experimental methodologies (supplemental Table S5) such as IP conditions, amount of cells or tissue used and the throughput of the experiment. Furthermore, the yields of quantified and identified peptides by MS may vary drastically between research labs; consequently, no fair comparisons could be conducted. Importantly, all the screened methodologies were found to have limited throughput because of lengthy (2–5 days) and laborious procedures.

We envisioned that reducing sample handling throughout all the purification steps would minimize peptide losses and significantly improve reproducibility. Thus, we designed a high-throughput 96-well plate format workflow for the simultaneous processing of tens of samples with commercially available reagents and consumables (Fig. 1). The platform employs a positive pressure processor which ensures a controlled and reproducible flow through the wells.

Briefly, tissue lysates are loaded on the first plate (Pre-clear plate in Fig. 1) containing Pro-A beads for clearance of endogenous antibodies, whereas cell lysates are loaded directly onto the plate (HLA class I plate in Fig. 1) containing anti-HLA-I antibodies covalently cross-linked to Pro-A beads for IP of HLA-I complexes. Lysates then drop directly from the first affinity plate onto the second plate (HLA class II plate in Fig. 1) that contains anti-HLA-II cross-linked to Pro-A beads. HLA class I and II plates are washed separately and each plate is then positioned on top of distinct C18 96-well plates (C18 solid phase extraction plate in Fig. 1). The HLA complexes are eluted from each of the affinity plates with TFA directly onto the corresponding C18 plate. After adequate washing of the C18 plates, the HLAp are eluted with ACN into collection plates and are ready to be dried by vacuum centrifugation and stored. The immunopurification procedure takes on average five hours including the desalting step and thus eliminates the in-process temporary storage of samples. To complement the immunopeptidomic analyses, total protein extracts and DNA can be collected from the investigated samples for shot-gun proteomics and genomics.

##### High-throughput Purification of HLA-Ip and HLA-IIp from Tissues and Cell Lines

**Fig. 2.**
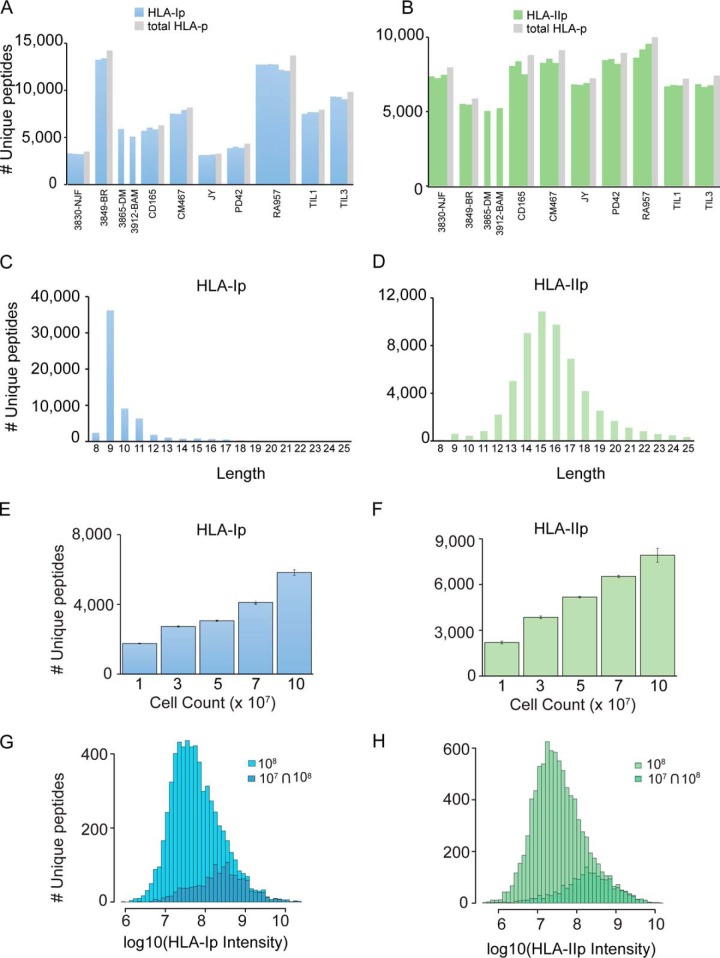
**In-depth and sensitive analysis of HLA-Ip and HLA-IIp at 1% FDR for peptide identifications.** *A*, Number of unique HLA-Ip (blue bars) and (*B*) HLA-IIp (green bars) identified for B- and T-cell lines and individual tissue samples, and in total (gray bars). *C*, Length distribution of HLA-Ip and (d) of HLA-IIp. *D*, Average number of HLA-Ip (blue bars) and (*E*) HLA-IIp (green bars) identified in triplicates in lysate volumes equivalent to 10, 30, 50, 70 and 100 -million CD165 cells. Data is represented as mean ± S.D. *F*, Distribution of intensities of HLA-Ip and (*G*) HLA-IIp detected in the samples of 100 million cells and those detected in samples of both 10 million and 100 million cells.

To assess the throughput and overall performance of our method, we first purified HLA-Ip and HLA-IIp in a single IP procedure (Plate 1) from a total of twenty one samples (with 3 additional mock samples), which included three replicates each from five B- and two T-cell lines (10^8^ cells per replicate). In a second experiment (Plate 2) we processed four primary meningioma tissues, using 0.7 to 1.47 grams per biological replicate. Detailed information about the experimental design is provided in supplemental Table S2 and clinical information and HLA typing are provided in supplemental Table S1. From plate number 1, a total of 42,556 unique HLA-Ip from 8975 source proteins and 43,702 unique HLA-IIp from 4501 source proteins were identified using a 1% peptide spectrum match FDR. The number of unique HLA-Ip in B- and T- cell lines varied from 3293 to 13,696 and from 7210 to 10,060 for HLA-IIp (Fig. 2*A*–2*B* and supplemental Table S6). Unlike the high concentration of about 15 mm used for carbamidomethylation of cysteines in shotgun proteomics workflows, the low concentration of 0.2 mm IAA in the IP lysis buffer facilitates irreversible inhibition of cysteine proteases, like caspases (51). Therefore, we identified a small percentage of on average 1.2% HLA-Ip and 1.9% HLA-IIp containing carbamidomethylated cysteines (supplemental Table S7). To exclude carry-over between wells during the affinity purification steps, we incorporated in plate 1 cross-linked beads not loaded with lysate (mock samples), and indeed no HLA-I or HLA-II complexes were detected here (supplemental Fig. S1*A*). In plate 2 we identified from 3497 to 14,213 HLA-Ip and from 5047 to 7972 HLA-IIp (at 1% FDR) from four patient-derived primary meningioma tissues (Fig. 2*A*–2*B* and supplemental Table S8).

##### In-depth and Accurate Immunopeptidomics Enables Determination of Consensus Binding Motifs

HLA-Ip datasets were highly enriched for ligands of typical length distribution for HLA-I (Fig. 2*C*). The consensus binding motifs of respective HLA-I alleles can be accurately de-convoluted from the identified peptides and the motifs match remarkably well to the known ones (7, 29, 52). However, in contrast to the HLA-I motifs, the core binding preferences of HLA-IIp are still poorly defined (36, 53). HLA-II molecules present longer peptides (mainly 12–19 mer and average a.a length of 15) (Fig. 2*D*) often sharing a binding core of typically 8–9 a.a. We anticipate that the great depth of our data will facilitate HLA-II motif determination. Similarly to HLA-I motif analysis (29), we de-convoluted the peptidomics data per sample and searched for the concordant motif between samples sharing the same HLA-DRB1 alleles (36). We further compared them to the motifs derived from assembled IEDB data (39). We were able to determine at least one HLA-DR motif in each of the samples with defined anchor residues typically located at positions 1, 4, 6, and 9. Furthermore, motifs of shared alleles showed a high degree of similarity between samples (supplemental Fig. S2).

##### Challenging the Sensitivity of the Immunopeptidomics Platform for Samples of Limited Amount

Sample amount availability poses a major limitation for the recovery of HLA class I and II peptides, especially in clinically relevant samples (12, 25–27). We reasoned that because of the fast recovery of HLA complexes and minimal sample handling, our method would also achieve substantial peptide yields even from samples of limited amount. Thus, we decided to challenge the sensitivity of our immunopurification platform by assessing HLA-I and -II peptide yields for decreasing cell amounts, down to 10^7^ B-cells. We selected a B-cell line (CD165) characterized with an average yield of peptides (from the B-cell lines analyzed). Lysate volumes equivalent to 10, 30, 50, and 70 million cells in triplicates were loaded on the plate. The linear recovery of the HLA heavy chains and β2m was visualized on a SDS-gel (supplemental Fig. S1*B*). Similarly, the unique HLA-Ip and -IIp identified linearly correlated with the amount of cells (Fig. 2*E*–2*F*). From as little as 10 million cells we identified a total of 1846 HLA-Ip and 2633 HLA-IIp peptides (Fig. 2*E*–2*F* and supplemental Tables S9 and S10) and as expected, the peptides identified from 10 million cells were among the most abundant ones detected in the samples containing a 100 million cells (Fig. 2*G*–2*H*).

##### Assessment of Intra- and Interplate Reproducibility

**Fig. 3.**
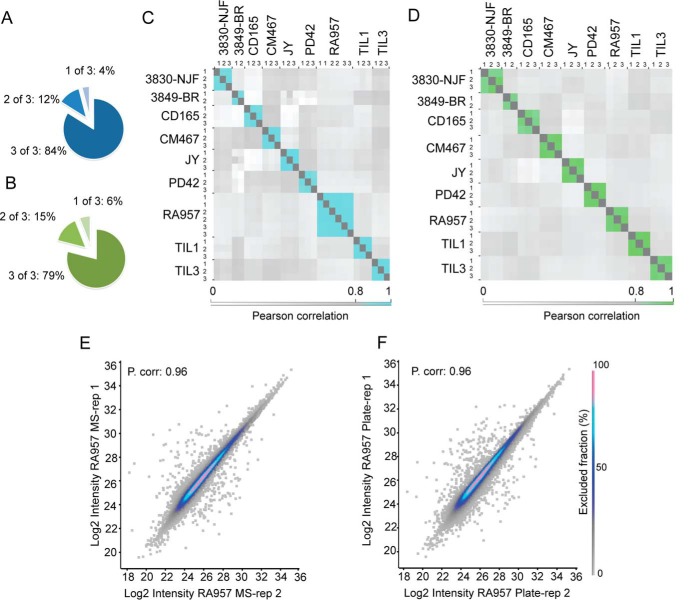
**Assessment of intra-plate reproducibility.** *A*, Overlap in the frequency of HLA-Ip and (*B*) HLA-IIp identified in three plate replicates of RA957 samples. *C*, Intra-plate reproducibility calculated by Pearson correlations of log2 transformed intensities of HLA-Ip and (*D*) HLA-IIp identified across the different MS measurements. *E*, Examples of comparative semi-quantitative analysis of HLA-Ip detected in two MS measurements (referred here as technical MS replicates) of one RA957 sample and (*F*) of two representative plate replicates of RA957 samples. Values of the Pearson correlation are indicated.

For a thorough evaluation of the reproducibility, we distributed the same amount of lysates from each of the B- and T-cell lines into triplicate wells within the same plate (Plate 1). First, we assessed the overlap of detection of HLA-Ip and HLA-IIp in one, two or all three replicates of the RA957 cell line. 84% of HLA-Ip overlapped in all 3 replicates, 12% in 2 out of 3 and only 4% in one replicate. In the case of HLA-IIp, 79% of peptides were found in all 3 replicates, 15% in 2 out of 3 and only 6% in one replicate (Fig. 3*A*–3*B*). The overall reproducibility of the MS signal at the peptide level displayed Pearson correlation coefficients (r) ranging from 0.89 to 0.98 for HLA-Ip, and from 0.89 to 0.97 for HLA-IIp (Fig. 3*C*–3*D*). Notably, the reproducibility between wells was as good as the reproducibility of MS-technical duplicates of the RA957 samples (Fig. 3*E*–3*F*). Additionally, CD165, CM647 and JY samples were distributed in Plate 1 (supplemental Table S2) to non-adjacent wells to assess how plate-positional effects would affect reproducibility; no evident plate-positional effects were observed (Fig. 3*C*–3*D*).

High correlations (r) of 0.93 were also observed between the peptides extracted from different sections of 3849-BR and 3830-NJF meningioma tissues (Fig. 3*C*–3*D*, see Plate 2, supplemental Table S2), emphasizing the applicability of our platform for more challenging clinical tissue samples. In addition, JY cells of similar amounts were purified on different days, with new reagents and using orthogonal wells across the plates to evaluate interplate performance (supplemental Tables S2 and S11). Average correlations (r) of 0.93 for HLA-Ip and 0.9 for HLA-IIp were observed (supplemental Fig. S3*A*–3*B*). The peptide recovery was further evaluated by spiking 15 heavy-labeled peptides into CD165 HLA-Ip samples. All 15 heavy-labeled and their endogenous counterparts were identified in each of the replicates. Their retention times are reported in supplemental Table S3. The CV of the ratio between the heavy-labeled and endogenous peptides was calculated for three exemplary cases resulting in a CV of 1% between the replicates (supplemental Table S4). The synthetic peptides were additionally used to evaluate carry-overs between wells during desalting steps and no synthetic peptides were detected in neighboring samples after manual inspection of the RAW MS data (supplemental Table S3).

##### Highly Reproducible Analysis Facilitates Label-free Comparative Study of the Drug-modulated Immunopeptidome

**Fig. 4.**
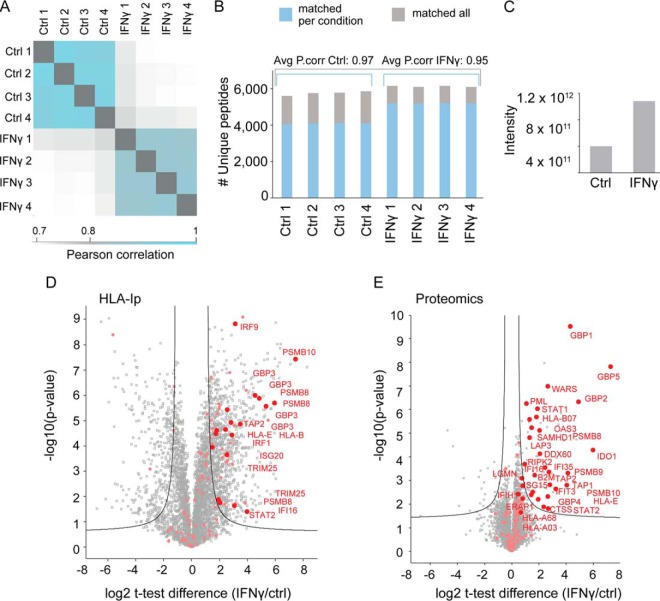
**Label-free semi-quantitative comparative analysis of IFNγ modulated immunopeptidome.** *A*, Reproducibility calculated by Pearson correlations of log2 transformed intensities from HLA-Ip of control and IFNγ -treated samples across the different MS measurements. *B*, Number of HLA-Ip identified from UWB.1 289 ovarian cancer cells untreated (control) and treated with IFNγ. The number of peptides identified with (gray) and without (blue) matching identifications across the treated and untreated samples and the average values of the Pearson correlations are indicated. *C*, Summed peptide intensities identified in each of the IFNγ treated and control samples. *D*, Volcano plot summarizing unpaired *t* test analysis of the immunopeptidome of IFNγ treated *versus* untreated cells. Peptides located above the lines are statistically significantly modulated in their level of presentation (FDR = 0.01, S0 = 1). All peptides derived from proteins related to immunity are highlighted in pink. Selected up-regulated peptides were highlighted in red, corresponding to well known intracellular mediators of IFNγ signaling. *E*, Volcano plot of unpaired *t* test analysis of the proteome of IFNγ treated *versus* untreated cells. Proteins located above the lines are statistically significantly modulated in their expression level (FDR = 0.01, S0 = 0.2). Selected proteins involved were similarly highlighted.

We reasoned that our streamlined method would enable a qualitative and quantitative assessment of HLAp alterations on external stimuli, potentially revealing the mechanistic mode of action. Thus, as a proof of concept we interrogated alterations induced by IFNγ on the UWB.1 289 ovarian cancer cell line. IFNγ is a key cytokine that activates multiple immune related signaling pathways and hence modulates the expression of hundreds of genes. Specifically, it is known to up-regulate the expression of HLA-I complexes as well as other key proteins involved in the antigen processing and presentation pathway in tumor cells (54). Indeed, on 24 h of IFNγ treatment of the UWB.1 289 cells, we detected enhanced cell surface expression of HLA-I by FACS and a global increase of total HLA-I and β2m by SDS-gel analysis (supplemental Fig. S4*A*–S4*B*). Average Pearson correlations of 0.95 between IFNγ-treated and of 0.97 between control replicates were obtained (Fig. 4*A*). We identified on average 4090 unique HLA-Ip in controls and 5195 peptides in IFNγ treated samples (Fig. 4*B*). However, with the “match between runs” option which enables the assignment of identifications to MS features that were not selected for fragmentation in all replicates (55), the number of identified peptides in controls evened up to the number detected in IFNγ treated samples (Fig. 4*B* and supplemental Table S12). The overlap of the two datasets was then as high as 91%. This observation, together with the sum of peptide signal intensities (Fig. 4*C*) suggested that IFNγ led to quantitative reshaping of the repertoire.

##### Immunopeptidomics and Proteomics Capture Similar Global Changes on IFNγ Treatment

We further explored qualitative global changes in the HLAp repertoire modulated on IFNγ treatment and detected 1157 HLA-Ip that were significantly up-regulated and 551 down-regulated HLA-Ip (*t* test FDR = 0.01, S0 = 1). Among the up-regulated HLA-Ip we detected peptides derived from well-known intracellular mediators of IFNγ (Fig. 4*D*) (56) and this observation was confirmed with our proteomics analysis (supplemental Table S13). Proteins involved in antigen processing and presentation and consequently in the IFN-mediated immune response, were significantly up-regulated, among them STAT1 and 2, TAP1 and 2, β2m, OAS3, WARS, IFI16, and IRF (Fig. 4*D*). The constitutive proteasomal subunits (*i.e.* β5 (PSMB5), β1 (PSMB6) and β2 (PSMB7)) were not found to be differentially regulated on IFNγ treatment. On the other hand, the immunoproteasomal subunits (*i.e.* β5i (PMSB8) β1i (PSMB9), and β2i (PSMB10)) were up-regulated on exposure to IFNγ in both peptidomics and proteomics datasets (Fig. 4*D*–4*E*), supporting the switch from the constitutive- to the immunoproteasome (57). Notably, the constitutive proteasome has both tryptic and chymotryptic-like activities, whereas the IFNγ-induced immunoproteasome has been shown to exhibit quantitatively higher chymotryptic-like activity (58).

##### IFNγ Induced Presentation of Longer Peptides and of Peptides Harboring C-terminal Chymotryptic-like Residues

**Fig. 5.**
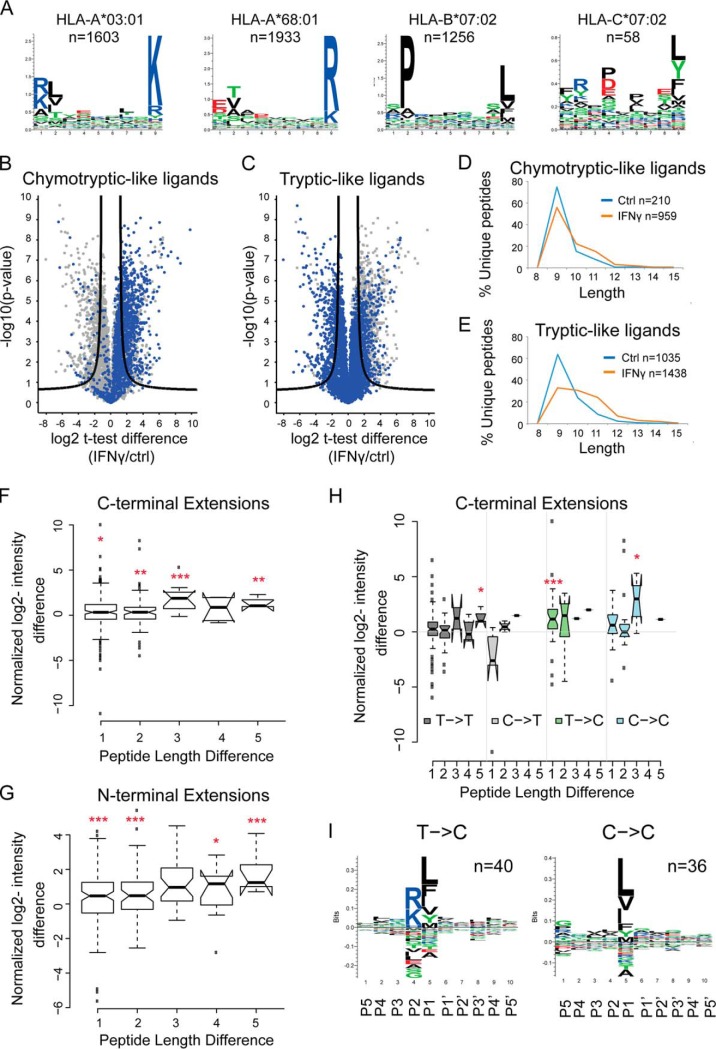
**Impact of IFNγ on global features of HLA class-I repertoire.** *A*, Peptides were assigned to the different HLA allotypes based on binding affinity predictions and their binding motifs depicted with sequence logos. *B–C*, Volcano plots summarizing unpaired *t* test analysis of the immunopeptidome of IFNγ treated *versus* untreated cells. Peptides located above the lines are statistically significantly modulated in their level of presentation (FDR = 0.01, S0 = 1). All chymotryptic-like (*B*) and tryptic-like ligands (*C*) were highlighted, respectively. *D–E*, Length distribution of peptides uniquely identified in IFNγ treated (orange) or control (blue) samples according to their chymotryptic- (*D*) or tryptic-like (*E*) properties. *F–G*, Intensity changes on IFNγ treatment were calculated for longer peptides against their shorter versions for both C- or N-terminal extensions: Normalized log2-intensity difference = log2((IFNγ_long_-ctrl_long_)/(IFNγ_short_-ctrl_short_)). (*H*) C-terminal nested versions were grouped based on whether their extended peptides remained tryptic-like (T→T), chymo-tryptic like (C→C), or if their specificities were switched (C→T or T→C). Log2-intensity changes on IFNγ treatment were calculated for longer peptides against their shorter versions (*I*) For T→C and C→C peptide pairs, the sequence logos around the cleavage site of the long peptides (C-terminal P1–5, downstream P'1–5) are depicted (one-sided *t* test, *p* value * < 0.1; ** < 0.05; and *** < 0.01).

As the chosen UWB.1 289 cell line expresses HLA-I alleles of C-terminal tryptic motifs (A03:01, A68:01) and C-terminal chymotryptic-like motifs (B07:02) (Fig. 5*A*) we hypothesized that analyzing the repertoire changes on IFNγ could uncover the impact of the immunoproteasome on the presented ligandome. First, we grouped the peptides based on their chymotryptic- or tryptic-like C-terminal (regardless of their HLA allele preferences). This revealed an enhanced presentation of chymotryptic-like ligands (Fig. 5*B*), whereas the presentation of tryptic-like ligands did not differ substantially on IFNγ treatment (Fig. 5*C*). Another global effect of the treatment was a general distribution of longer peptides that were uniquely detected on IFNγ treatment compared with those in control samples (Fig. 5*D*–5*E*).

Previous reports have shown peptides containing the binding motif within a common core region but extending beyond the motif in either N- or C-terminal directions (21, 42, 59, 60). About 7% of the peptides in this dataset were found overlapping entirely with longer peptide sequences. When the short and long peptide pairs were found to start at the same position we named them C-terminal elongated pairs. Similarly, short and long peptide pairs ending at the same position were named N-terminal elongated pairs (supplemental Table S14). We observed that after IFNγ stimulation, the longer peptides, in both N- and C-terminal directions, were often significantly more abundant compared with their shorter counterparts (Fig. 5*F*–5*G*), thereby contributing to the global length shift (Fig. 5*D*–5*E*). The extensions range between one to five a.a. along both termini.

Because the proteasome is known to determine the C-terminal cleavage specificity of HLA-Ip, we further explored if the enhanced presentation of the C-terminal elongated peptides is in agreement with the global increase in presentation of chymotryptic-like peptides on IFNγ treatment. Thus, we grouped the C-terminal elongated pairs based on whether their C-terminal remained tryptic-like, chymotryptic-like, or if their specificity was switched. We observed that on treatment, elongated chymotryptic-like peptides were significantly more abundant than their shorter tryptic-like peptides, mainly by one a.a. (supplemental Table S14 and Fig. 5*H*). The switch in cleavage specificity can be further visualized by comparing long peptides changing from tryptic- to chymotryptic-like (*n* = 40) and those maintaining their chymotryptic-like (*n* = 36) C-terminal cleavage specificity (Fig. 5*I*).

##### Allele-specific Analysis of the Immunopeptidome

Allele-specific presentation on IFNγ can be defined by other factors apart from the immunoproteasomal cleavage preferences. Therefore, we predicted the binding affinities with NetMHC 4.0 for HLA-A*03:01, -A*68:01, -B*07:02 and -C*07:02 (Fig. 5*A* and supplemental Table S15). We could not predict binding to the HLA-C*03:32 allele as the motif for this allele is currently still unknown and we excluded the HLA-C*07:02 binders because of their small population size (*n* = 58). Finally, we assigned the allele specificities to peptides that were predicted to bind to only one allele.

In line with the analysis for chymotryptic-like ligands, we detected an enhanced presentation of the HLA-B*07:02 peptides which contains C-terminal chymotryptic-like a.a. (Fig. 5*B* and supplemental Fig. S4*C*). Increased expression of HLA-B*07:02 heavy chain molecules was evident also at the proteomic level (Fig. 4*E*). Both HLA-A*03:01 and -A*68:01 have tryptic-like binding motifs, but with marked differences at the C-terminal (Fig. 5*A*). When examining these allele-specific populations, we observed that peptides predicted to bind the HLA-A*03:01 molecules were slightly down-regulated in contrast to HLA-A*68:01 ones (supplemental Fig. S4*C*). Overall, the predicted binding affinities of HLA-B*07:02 ligands were similar regardless of IFNγ treatment, whereas HLA-A*03:01 and -A*68:01 ligands were predicted to bind with higher affinity on IFNγ stimulation, although not statistically significant in all peptide lengths (supplemental Fig. S5*A*). We detected a statistically significant increase in the composition of hydrophobic a.a. (as indicated by the hydrophobicity score Φ, supplemental Fig. S5*B*) in peptides across all three alleles.

## DISCUSSION

The extraction procedure of HLA ligands for deep MS analysis has been a major limitation (19). We present here a greatly improved IP-based HLA-Ip and -IIp purification pipeline which has been rigorously optimized and encompasses several new features and advantages: (1) the fast IP step minimizes artifacts possibly introduced during long incubations, (2) minimal in-process sample handling and freezing steps allow competitive recovery and sensitivity, (3) drastic reduction in the amount of expensive antibody-crosslinked beads, (4) parallel processing of dozens of samples and (5) elimination of error prone steps, making the pipeline suitable for processing valued patient-derived tissue samples. We demonstrated the high-throughput nature of the workflow by purifying in a single IP procedure HLA-Ip and HLA-IIp from twenty one samples (only 10^8^ cells per replicate). The depth and reproducibility of the enriched HLA-I and -II peptidomes were outstanding with an average of more than 7500 unique peptides identified in single IP for both class I and II (Fig. 2*A*–2*B*). Furthermore, the overall reproducibility (Pearson correlation coefficient) ranged from 0.89 to 0.98 for HLA-Ip, and from 0.89 to 0.97 for HLA-IIp (Fig. 3*C*–3*D*). We affirmed the pipeline's robust performance and clinical applicability by parallel processing of four primary meningioma tissues of different quantities, which matched well with their peptide yields (Figs. 2*A*–2*B* and 3*C*–3*D*).

Importantly, a major bottle-neck of immunopeptidomics is still the requirement of a relatively large amount of cells or tissue material, which is not always feasible to obtain from clinical samples. We showed that our methodology reached a degree of sensitivity that enables us to identify 1846 HLA-Ip and 2633 HLA-IIp from as little as 10 million cells (Fig. 2*E*–2*F*). This achievement therefore highlights the possibility to drastically scale down the sample amount, when required.

The purity and depth of the extracted peptidomes allowed us to determine high-resolution HLA class II motifs comparable to the IEDB data (supplemental Fig. S2). A current limitation to obtain the HLA class II binding motifs from this approach arises when the alleles from an individual sample share very similar binding motifs (*i.e.* DRB1*01:01 and DRB1*07:01). In such eventuality, GibbsCluster cannot cluster the peptides into distinct motifs. Motif annotation can also be hindered by the lack of existing data and reference motifs in the IEDB database.

The current data allowed the identification of motifs for most of the HLA-DR alleles present in each sample. However, these samples also contained HLA-DP and HLA-DQ alleles. As such we cannot exclude that some of the motifs predicted by Gibbscluster for which we could not find any HLA-DR may correspond to -DP or -DQ motifs. Unfortunately, both the alpha and beta chains show high variability in these alleles, such that each sample could contain up to four different HLA-DP and four different HLA-DQ combinations, which makes motif identification and annotation much more challenging. Our work suggests that samples expressing mono-allelic selected HLA-DP and HLA-DQ alleles should be explored to reveal their specificities. We anticipate that with the growing number of samples analyzed with the method described in this work, the number of newly identified HLA-II binding motifs will quickly grow and, re-interrogation of this data can be of use for improving HLA-II ligand predictions in machine learning studies.

The development of a reproducible and high-throughput methodology allows us to generate high quality data to gain more insight into biological questions. Thus, as a proof of concept we demonstrated the feasibility of performing comparative screening on IFNγ treatment. Exposure of tumor cells to IFNγ is known to induce pro-inflammatory gene signatures that consequently lead to enhanced recognition by cytotoxic T-lymphocytes (28, 61) mediated by the up-regulation of the APPM and HLA surface expression. Augmented surface presentation of HLA complexes could lead to a higher probability of presentation and, hence, recognition of immunogenic epitopes. However, no high-quality mapping of the IFNγ modulated peptidome has been reported so far and therefore, the overall properties of the presented repertoire on stimulation remain unknown.

Here, we uncovered a global modulation in the immunopeptidome on exposure to IFNγ. We estimated that the HLA-Ip repertoire increased by 170%, as depicted from the differential MS intensities (Fig. 4*C*). The boost in HLA-I expression was also validated by FACS, as well as by semi-quantification of the HLA heavy chains and β2m (supplemental Fig. S4*A*–S4*B*). In addition, the proteomics analysis confirmed the up-regulation of intracellular sensors and mediators of the IFNγ signaling pathway as well as several of its effectors (Fig. 4*E*), as previously reported (56). The IFNγ signature was clearly conveyed at the peptide repertoire (Fig. 4*D*–4*E*), supporting the reported positive correlation between proteome expression and antigen presentation (7). In fact, both at the peptidome and proteome level, various key components of the APPM were up-regulated, such as TAP 1 and 2, β2m, HLA heavy chains and the immunoproteasomal subunits (*i.e.* β5i (PMSB8) β1i (PSMB9), and β2i (PSMB10)). Our results clearly showed a marked shift in presentation of chymotryptic-like ligands (*i.e.* HLA-B*07:02 binders) on IFNγ stimulation (Fig. 5*A* and supplemental Fig. S4*C*). This can be explained by the combined effect of the increased expression of HLA-B*07:02 molecules (Fig. 4*E*) and by the switch from the constitutive proteasome to immunoproteasome. In fact, proteasomal switching may have led to a more efficient generation of peptides harboring C-terminal chymotryptic-like residues (*i.e.* same as HLA-B*07:02 binders) (58), whereas the overall tryptic-like ligand presentation remain largely unchanged (Fig. 5*B*–5*C* and supplemental Fig. S4*C*). However, when allele specificities were taken into account, the presentation of HLA*03:01 and A*68:01 binders were differentially regulated on treatment possibly because of subtle proteasomal (or other peptidases) cleavage preferences or to the slightly different expression levels of these HLA alleles (supplemental Fig. S4*C*).

A general tuning of the APPM toward presentation of longer peptides was globally detected (Fig. 5*D*–5*E*). These longer peptides may bind via canonical residues facilitated by the bulging of the middle part of the peptide or they could bind with inner anchors leaving the extension to protrude from one end of the binding groove (42, 60, 62, 63). Uniquely to our study, we were able to quantitatively assess the enhanced presentation of peptides varying in length on IFNγ stimulation. N-terminal extended peptides did not show cleavage patterns; this is expected because of the downstream trimming events that takes place in the ER. N-terminal extended versions of canonical peptides still may contain appropriate HLA binding motifs (59). Intriguingly, we also detected C-terminal tryptic-like peptides that have statistically significant enriched chymotryptic-like longer versions (one a.a.), possibly hinting toward an enhanced chymotryptic-like activity of the immunoproteasome (Fig. 5*H*). We speculate that the production of longer peptides may have been favored, not only by proteasome switching but also by TAP shuttling (64), which was also significantly up-regulated in our proteomic analysis. Interestingly, it was observed that the first three N-terminal residues and the C-terminal residue were the most critical for TAP-binding (65). Therefore, the longer peptides we detected may have been favored because of their specific physicochemical properties for binding to the TAP (64).

Altogether, in this proof of concept study, we shed light on the quantitative re-shaping of the presented peptidome imposed by IFNγ treatment. Further investigation across additional cell lines covering more HLA allotypes is required to precisely determine the exact molecular mechanisms underlying these changes and if the observed modulation of the peptidome can be generalized. Further research is also needed to reveal the cellular conditions that facilitate the generation and surface presentation of peptides of higher quality in terms of fitness to the HLA alleles, such as binding affinity, sequence hydrophobicity and peptide length. This would enable the incorporation of additional features in prediction algorithms of antigen presentation and hence improve their accuracy (43, 66). We present a robust methodology that enables LFQ comparative immunopeptidomics applicable for the investigation of perturbations in the antigen presentation caused for example by pathogenic infections, autoimmunity and cancer. Several pioneering vaccine companies and research labs have recently incorporated MS-based immunopeptidomics as a discovery tool to gain knowledge of the presented peptidome for improved binding predictions or to directly identify mutated antigens and clinically relevant targets for vaccinations (67, 68). We hope that our method will accelerate the development of vaccines and will assist in implementing this technology in clinical practice.

## DATA AVAILABILITY

All the RAW data, the FASTA reference file, MaxQuant parameters and output tables, including selected MaxQuant output tables used for analyses (filtered for contaminants and reverse hits) have been deposited to the ProteomeXchange Consortium (69) via the PRIDE partner repository with the dataset identifier PXD006939.

## Supplementary Material

Supplemental Data

## Footnotes

^1^ The abbreviations used are:

HLA-I: human leukocyte antigen class I
HLAp: HLA-bound peptides
a.a.: amino acid
APPM: antigen processing and presentation machinery
β2m: beta-2-microglobulin
CV: coefficient of variation
FA: formic acid
FDR: false discovery rate
HLA: human leukocyte antigen
HLA-II: human leukocyte antigen class II
HCD: higher-energy collision dissociation
HCl: hydrochloric acid
IEDB: immune epitope database
IFNγ: Interferon gamma
IP: immunoaffinity purification
LFQ: label-free quantification
NaCl: sodium chloride
NH_4_AcO: ammonium acetate
Pro-A: protein-A sepharose 4B
SCX: strong-cation-exchange
MS: mass spectrometry
FBS: fetal bovine serum
TIL: tumor infiltrating lymphocyte
IAA: iodoacetamide
ACN: acetonitrile
TFA: trifluoroacetic acid
AMBIC: ammonium bicarbonate.
