# Supplementary material for: High-throughput and Sensitive Immunopeptidomics Platform Reveals Profound Interferonγ-Mediated Remodeling of the Human Leukocyte Antigen (HLA) Ligandome
Source: Mol Cell Proteomics. 2017 Dec 14;17(3):533–48. doi: 10.1074/mcp.TIR117.000383 (PMC5836376; doi:10.1074/mcp.TIR117.000383)
Supplement: Supplemental Data [file supp_17_3_533__index.html]

High-throughput and sensitive immunopeptidomics platform reveals profound IFNγ-mediated remodeling of the HLA ligandome — High-throughput and sensitive immunopeptidomics platform — High-throughput and Sensitive Immunopeptidomics Platform Reveals Profound Interferonγ-Mediated Remodeling of the Human Leukocyte Antigen (HLA) Ligandome — High-throughput and Sensitive Immunopeptidomics Platform — Supplemental Data 

# High-throughput and Sensitive Immunopeptidomics Platform Reveals Profound Interferonγ-Mediated Remodeling of the Human Leukocyte Antigen (HLA) Ligandome

## Supplemental Data

- Supplemental information and figures - Supplemental information and figures
- Supplemental Table S1 - Supplemental Table S1
- Supplemental Table S2 - Supplemental Table S2
- Supplemental Table S3 - Supplemental Table S3
- Supplemental Table S4 - Supplemental Table S4
- Supplemental Table S5 - Supplemental Table S5
- Supplemental Table S6 - Supplemental Table S6
- Supplemental Table S7 - Supplemental Table S7
- Supplemental Table S8 - Supplemental Table S8
- Supplemental Table S9 - Supplemental Table S9
- Supplemental Table S10 - Supplemental Table S10
- Supplemental Table S11 - Supplemental Table S11
- Supplemental Table S12 - Supplemental Table S12
- Supplemental Table S13 - Supplemental Table S13
- Supplemental Table S14 - Supplemental Table S14
- Supplemental Table S15 - Supplemental Table S15
