# Supplementary material for: High-throughput and Sensitive Immunopeptidomics Platform Reveals Profound Interferonγ-Mediated Remodeling of the Human Leukocyte Antigen (HLA) Ligandome
Source: Mol Cell Proteomics. 2017 Dec 14;17(3):533–48. doi: 10.1074/mcp.TIR117.000383 (PMC5836376; doi:10.1074/mcp.TIR117.000383)

Supplemental Data for:

High-throughput and sensitive immunopeptidomics platform reveals profound IFNγ-mediated remodeling of the HLA ligandome

Chloe Chong[1,2]*, Fabio Marino[1,2]*, HuiSong Pak[1,2], Julien Racle[1,2,5], Roy T. Daniel[3], Markus Müller[4], David Gfeller[1,2,5], George Coukos[1,2], Michal Bassani-Sternberg[1,2#]

[1] Ludwig Institute for Cancer Research, University of Lausanne, 1066 Epalinges, Switzerland.

[2] Department of Oncology, University of Lausanne, 1015 Lausanne, Switzerland.

[3] Service of Neurosurgery, University Hospital of Lausanne, 1015 Lausanne, Switzerland.

[4] Vital IT, Swiss Institute of Bioinformatics, 1015 Lausanne, Switzerland.

[5] Swiss Institute of Bioinformatics, 1015 Lausanne, Switzerland.

[#] Correspondence author: Michal Bassani-Sternberg (Michal.bassani@chuv.ch)

*Equally contributing authors

Supplemental Figure Legends

Supplemental Fig. S1. SDS-gel semi-quantification of recovered subunits of the HLA complexes. (A) Eluted HLA-I heavy chains and β2m molecules, and HLA-II heavy chains from lysates of selected cell line samples and the three mock samples, respectively. (B) Eluted HLA-I heavy chains and β2m molecules, and HLA-II heavy chains for lysate volumes corresponding to 10, 30, 50, 70 and 100 million CD 165 B-cells, respectively.

Supplemental Fig. S2. Motif analyses of HLA-II immunopeptidomes. Motifs obtained by GibbsCluster for the various samples based on the new MS data and the motifs built from IEDB data corresponding to the HLA-DRB1 alleles present in each sample. The number of motifs plotted for each sample is the best number as determined by GibbsCluster. Numbers above each motif indicate the number of unique peptides assigned to it. Background colors and title above each motif indicate which HLA-DRB1 alleles was assigned to the motifs. When binding motifs are redundant, both alleles were assigned to the observed motifs.

Supplemental Fig. S3. Excellent inter-plate reproducibility. (A) Inter-plate reproducibility calculated by Pearson correlations of Log2 transformed intensities of HLA-Ip and (B) HLA-IIp purified from JY cells on different days and with different stocks of reagents and plates.

Supplemental Fig. S4. Increased expression of HLA-I complexes and allele-specific changes upon IFNγ treatment. (A) Increased expression of HLA-I on the surface of UWB.1 289 cells upon IFNγ treatment detected by FACS analysis. (B) SDS-gel semi-quantitative analysis confirmed global increase in intensities of HLA class I heavy chains and β2m molecules after IFNγ treatment. (C) Volcano plot summarizing unpaired t-test analysis of the immunopeptidome of IFNγ treated versus untreated cells. Peptides located above the lines are statistically significantly modulated in their level of presentation (FDR=0.01, S0=1). Peptides

were colored on the exact volcano plot as follows: peptides predicted to bind the HLA-B*07:02 in orange, to the HLA-A*03:01 in green and to the HLA-A*68:01 in blue.

Supplemental Fig. S5. Physicochemical properties of HLA-Ip upon IFNγ treatment. (A) Peptides were assigned to the different HLA allotypes and peptides uniquely identified in IFNγ treated (blue) or control (orange) samples were plotted for their distribution in predicted binding affinities. (B) IceLogo was used to calculate the statistics to find over- represented amino acids in each position of HLA-B*07:02, -A*68:01 and –A*03:01 predicted binders of the IFNγ dataset compared to the control. A difference of hydrophobicity scores (Φ) between IFNγ dataset compared to the control is reported together with their statistical significance. (unpaired t-test, p-value'*<0.1, ** <0.05 and *** <0.01).

Supplemental Table Legends

Supplemental Table S1. Description of samples. HLA typing are provided for each sample including clinical information, where relevant.

Supplemental Table S2. Experimental design. Information on the experimental design includes sample name, type of replicate, HLA purification type, sample size, experiment number, MS injection amount and name of the RAW file.

Supplemental Table S3. Heavy labelled synthetic peptides for validation of workflow performance. Detailed MS/MS information about the 15 isotopically heavy labeled synthetic peptides used as spiked-in standards are provided for the assessment of reproducibility and carry-over during the HLA-I and –II IP procedure.

Supplemental Table S4. Heavy labelled synthetic peptides for technical reproducibility assessment. Detailed MS/MS information about 3 selected isotopically heavy labeled synthetic peptides and their light counterparts were used to measure technical reproducibility between the three replicates. Area under the curve, AUC; standard deviation, SD; coefficient variation, CV.

Supplemental Table S5. Literature on HLA-I and HLA-II immunopeptidomics. Reviewed reports on the IP workflows for HLA-I and HLA-II immunopeptidomics were compared based on their published detailed protocol descriptions.

Supplemental Table S6. Peptide output table Experiment Plate Number 1. A list of HLA-I and –II peptides identified by MaxQuant from the “peptides” output table filtered for known contaminants and reverse. Plate Number 1 includes seven B- and T- cell lines that were processed in parallel.

Supplemental Table S7. Cysteine carbamidomethylated HLA peptides from Experiment Plate Number 1 and 2. A list of HLA-I and –II modified peptides identified by MaxQuant from the “modificationSpecificPeptides” output table filtered for known contaminants and reverse.

Supplemental Table S8. Peptide output table Experiment Plate Number 2. A list of HLA-I and –II peptides identified by MaxQuant from the “peptides” output table filtered for known contaminants and reverse. Plate Number 2 includes the parallel processing of four patient-derived meningioma tissues samples.

Supplemental Table S9. Peptide output table HLAIp Sensitivity Experiment. A list of HLA-I peptides identified by MaxQuant from the “peptides” output table filtered for known contaminants and reverse. The CD 165 B-cell line was used to assess the limits of sensitivity of our workflow with cell amounts ranging from 10-100 Million.

Supplemental Table S10. Peptide output table HLAIIp Sensitivity Experiment. A list of HLA-II peptides identified by MaxQuant from the “peptides” output table filtered for known contaminants and reverse. The CD 165 B-cell line was used to assess the limits of sensitivity of our workflo with cell amounts ranging from 10-100 Million.

Supplemental Table S11. Peptide output table JY Interplate Performance. A list of HLA-I and –II peptides identified by MaxQuant from the “peptides” output table filtered for known contaminants and reverse. HLA peptides from JY B-cells were purified on different days with different reagents to assess the interplate performance of our extraction procedure.

Supplemental Table S12. Peptide output table IFNγ Experiment. A list of HLA-I peptides identified by MaxQuant from the “peptides” output table filtered for known contaminants and reverse. HLA peptides were extracted from an ovarian cancer cell line upon IFNγ treatment.

Supplemental Table S13. Protein groups output table IFNγ Experiment. A list of proteins identified by MaxQuant from the "ProteinGroups" output table filtered for only identified by site, known contaminants and reverse from an ovarian cancer cell line upon IFNγ treatment.

Supplemental Table S14. N- and C-terminal elongated HLA-Ip pairs extracted from the normalized "peptides" output table of the IFNγ Experiment. Features such as the direction (N- or C-terminal) of elongation, length of elongation, C-terminal cleavage specificities and their normalized intensities before and after treatment are reported here for each peptide pair for the analysis of N- and C-terminal peptide pairs.

Supplemental Table S15. Peptide output table IFNγ Experiment with t-test values and predicted affinities. A list of HLA-I peptides identified by MaxQuant from the "peptides" output table filtered for known contaminants and reverse. HLA peptides were extracted from an ovarian cancer cell line upon IFNγ treatment. The values were log2 transformed, normalized and imputed. Unpaired two-sided t-test (FDR: 0.01, S0: 1) was performed between IFNγ and ctrl groups. HLA specificities were assigned for peptides that were predicted to bind one allele only.

Supplemental Fig. S1

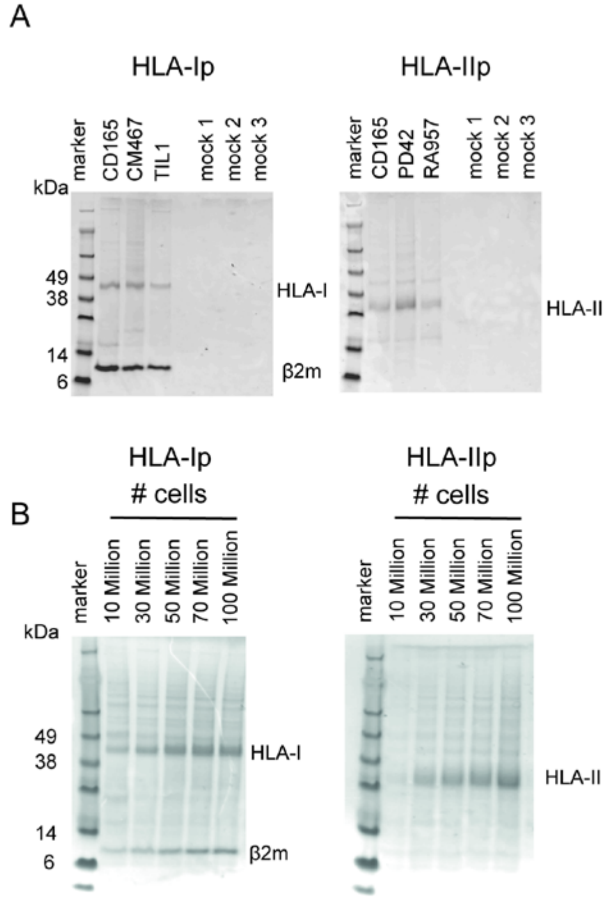

Supplemental Fig. S2.

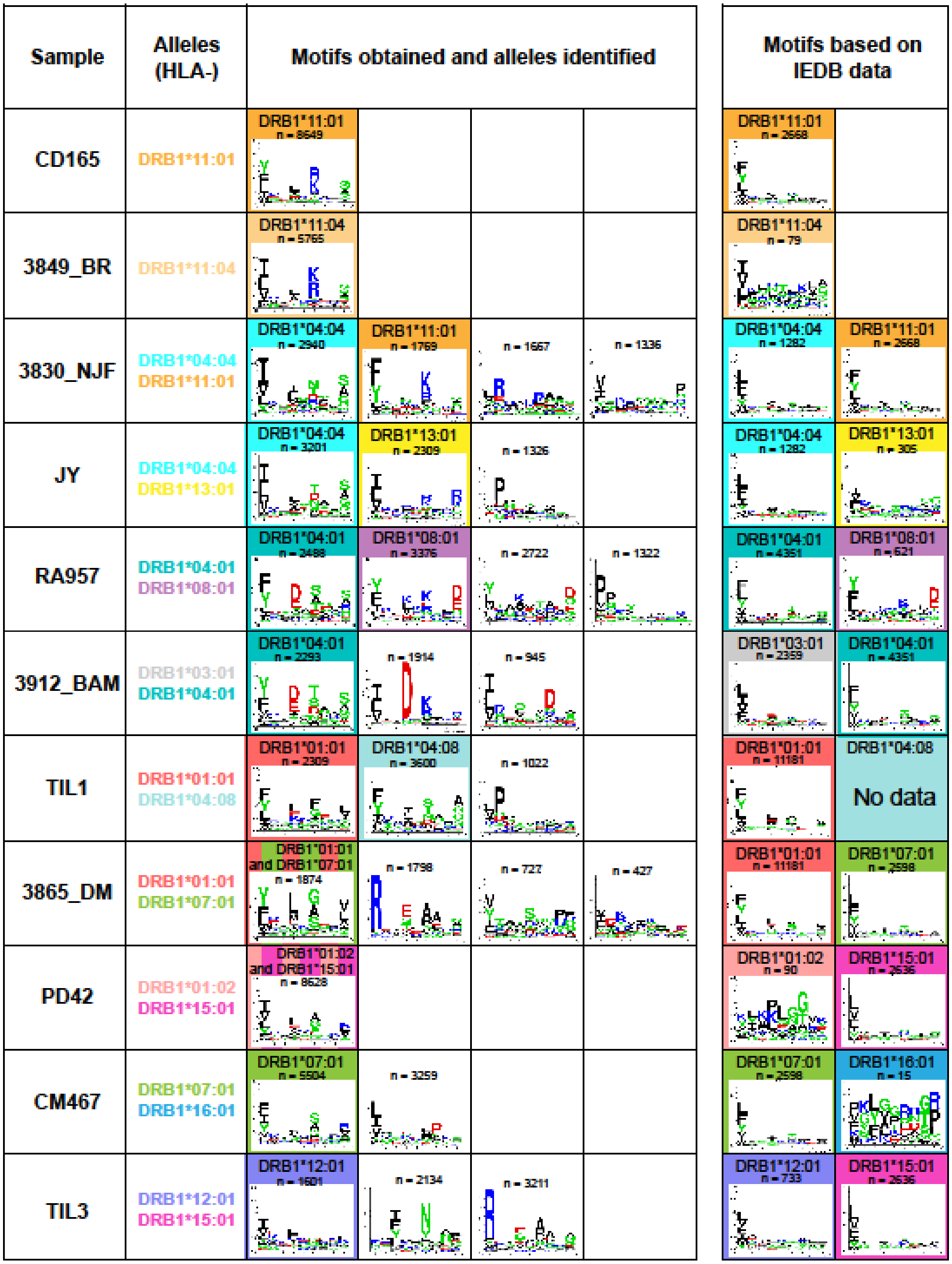

Supplemental Fig. S3.

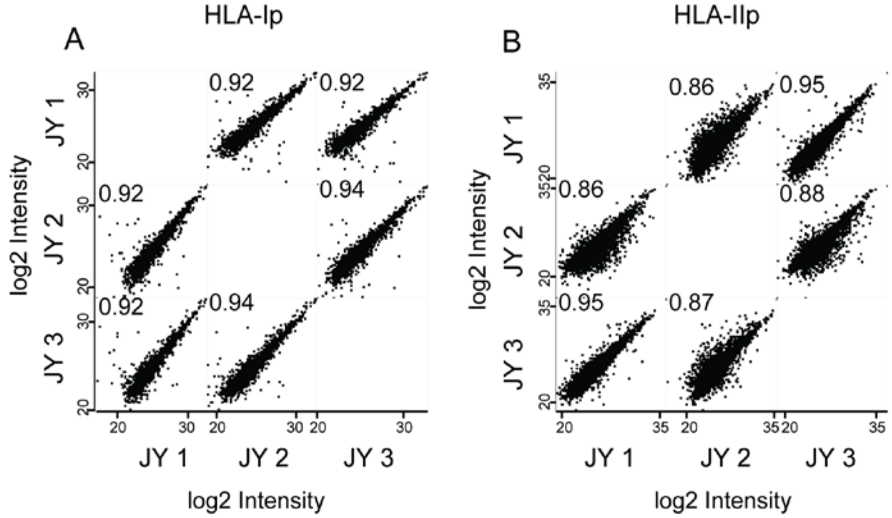

Supplemental Fig. S4.

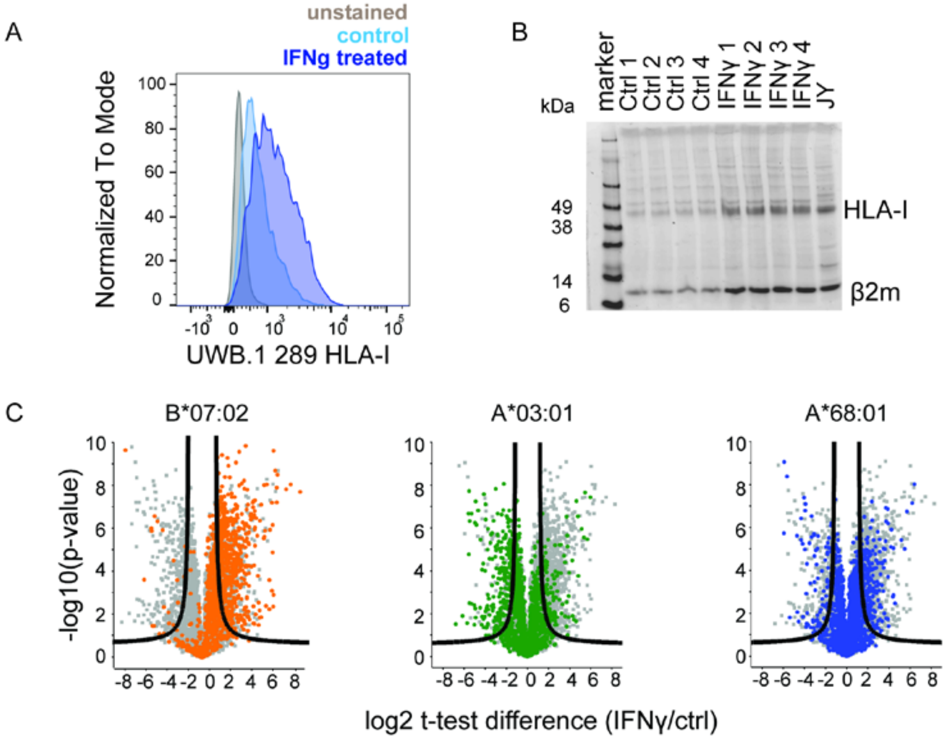

Supplemental Fig. S5.

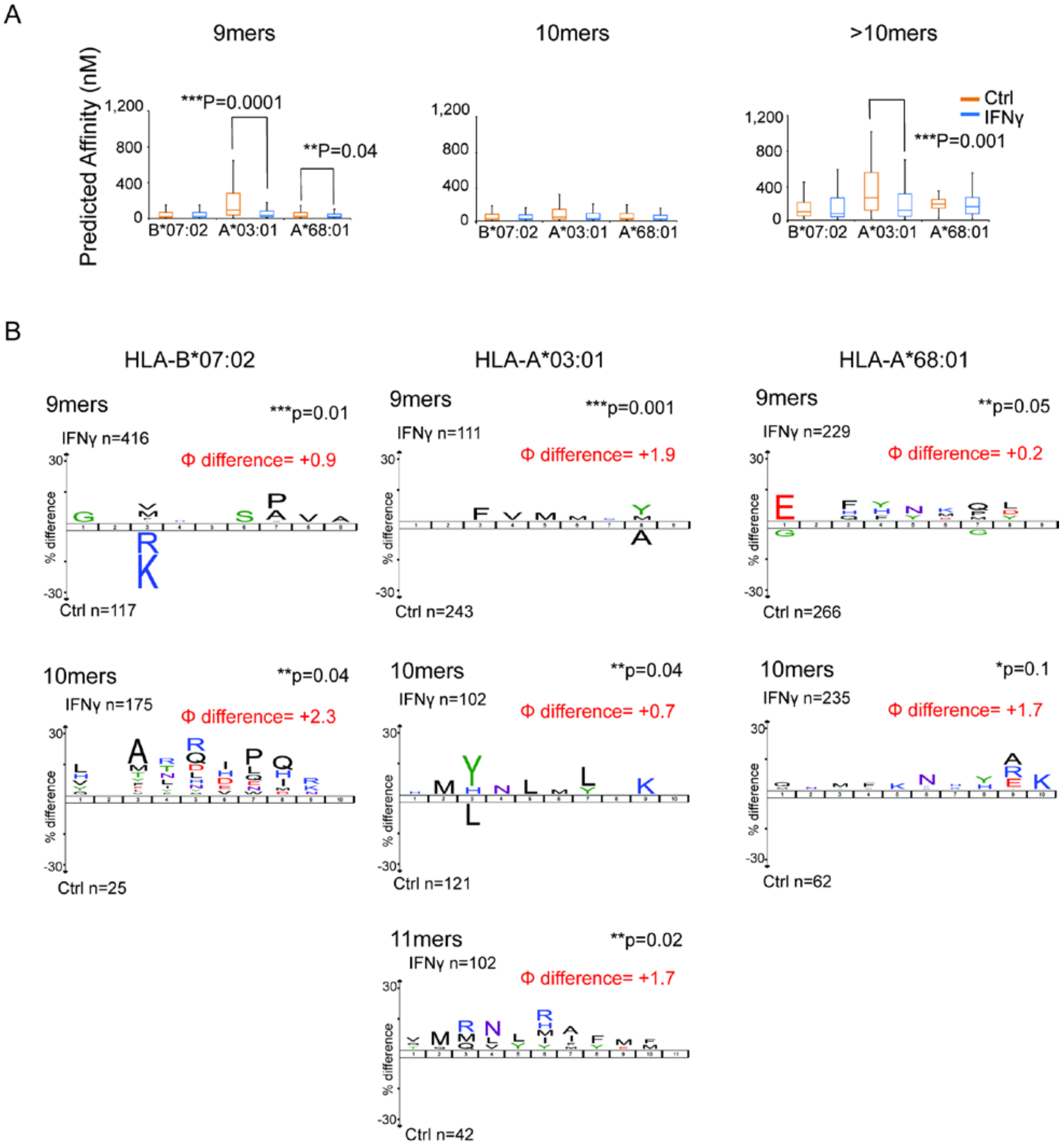

Supplement: Supplemental Data [file supp_TIR117.000383_132937_2_supp_37775_p0vb9k.pdf]
