# Supplementary material for: High-throughput and Sensitive Immunopeptidomics Platform Reveals Profound Interferonγ-Mediated Remodeling of the Human Leukocyte Antigen (HLA) Ligandome
Source: Mol Cell Proteomics. 2017 Dec 14;17(3):533–48. doi: 10.1074/mcp.TIR117.000383 (PMC5836376; doi:10.1074/mcp.TIR117.000383)
Supplement: Supplemental Data [file supp_TIR117.000383_132937_2_supp_37773_p0v9pn.pdf]

Table S1. Clinical information and HLA typing.

	Clinical information		HLA A		HLA B		HLA C		HLA DQB1		HLA DRB1	
	Tumor; grade	Age; Gender	1st allele	2nd allele	1st allele	2nd allele	1st allele	2nd allele	1st allele	2nd allele	1st allele	2nd allele
**CD165**	-	-	A*02:05	A*24:02	B*15:01	B*50:01	C*03:03	C*06:02	DQB1*03:01	DQB1:03:01	DRB1*11:01	DRB1*11:01
**CM647**	-	-	A*01:01	A*24:02	B*13:02	B*39:06	C*06:02	C*12:03	DQB1*02:02	DQB1*05:02	DRB1*07:01	DRB1*16:01
**PD 42**	-	-	A*02:06	A*24:02	B*07:02	B*55:01	C*01:02	C*07:02	DQB1*05	DQB1*06	DRB1*01:02	DRB1*15:01
**RA957**	-	-	A*02:20	A*68:01	B*35:03	B*39.01	C*04:01	C*07:02	DQB1*03:01	DQB1*04:02	DRB1*04:01	DRB1*08:01
**TIL1**	-	-	A*02:01	A*02:01	B*18:01	B*38:01	C*05:01	C*05:01	DQB1*03:01	DQB1*05:01	DRB1*01:01	DRB1*04:08
**TIL3**	-	-	A*01:01	A*23:01	B*07:02	B*15:01	C*12:03	C*14:02	DQB1*03:01	DQB1*05:02	DRB1*12:01	DRB1*15:01
**3830-NJF**	Meningioma;1	45;M	A*02:01	A*03:01	B*07:02	B*07:02	C*07:02	C*07:02	DQB1*03:01	DQB1*03:02	DRB1*04:04	DRB1*11:01
**3849-BR**	Meningioma;3	65;M	A*01:01	A*31:01	B*38:01	B*44:02	C*05:01	C*12:03	DQB1*03:01	DQB1*03:01	DRB1*11:04	DRB1*11:04
**3912-BAM**	Meningioma;2	63;F	A*01:01	A*29:06	B*08:01	B*15:01	C*03:04	C*07:01	DQB1*02:01	DQB1*03:02	DRB1*03:01	DRB1*04:01
**3865-DM**	Meningioma;1	50;F	A*01:01	A*03:01	B*35:01	B*57:01	C*04:01	C*06:02	DQB1*03:03	DQB1*05:01	DRB1*01:01	DRB1*07:01
**UWB.1 289 mut**	-	-	A*03:01	A*68:01	B*07:02	B*07:02	C*03:32	C*07:02	n.a	n.a	n.a	n.a
***JY**	-	-	A*02:01	A*02:01	B*07:02	B*07:02	C*07:02	C*07:02	HLA DQA1*30:11	HLADPB1*02:01	DRB1*04:04	DRB1*13:01

* From the European Collection of Authenticated Cell Cultures (ECACC), http://www.phe-culturecollections.org.uk
