# Supplementary material for: High-throughput and Sensitive Immunopeptidomics Platform Reveals Profound Interferonγ-Mediated Remodeling of the Human Leukocyte Antigen (HLA) Ligandome
Source: Mol Cell Proteomics. 2017 Dec 14;17(3):533–48. doi: 10.1074/mcp.TIR117.000383 (PMC5836376; doi:10.1074/mcp.TIR117.000383)
Supplement: Supplemental Data [file supp_TIR117.000383_132937_2_supp_37764_p0v9mk.pdf]

Table S2. Experimental design

Sample Name	Type of replicate			HLA	Cell Count	Plate Exp. Number	Well Position	Injection Amount	Raw file name
RA957	BR	PR	MSR	HLA-Ip	1.00E+08	1	A1	1/3	20170302_QEh1_LC2_FaMa_ChCh_SA_HLApI_RA957_1_R1
			MSR	HLA-Ip	1.00E+08	1	A1	1/3	20170302_QEh1_LC2_FaMa_ChCh_SA_HLApI_RA957_1_R2
		PR	MSR	HLA-Ip	1.00E+08	1	A2	1/3	20170302_QEh1_LC2_FaMa_ChCh_SA_HLApI_RA957_2_R1
			MSR	HLA-Ip	1.00E+08	1	A2	1/3	20170302_QEh1_LC2_FaMa_ChCh_SA_HLApI_RA957_2_R2
		PR	MSR	HLA-Ip	1.00E+08	1	A3	1/3	20170302_QEh1_LC2_FaMa_ChCh_SA_HLApI_RA957_3_R1
			MSR	HLA-Ip	1.00E+08	1	A3	1/3	20170302_QEh1_LC2_FaMa_ChCh_SA_HLApI_RA957_3_R2
CD165	BR	PR		HLA-Ip	1.00E+08	1	A4	1/3	20170303_QEh1_LC2_FaMa_ChCh_SA_HLApI_CD165spike_1_R1
		PR		HLA-Ip	1.00E+08	1	B2	1/3	20170303_QEh1_LC2_FaMa_ChCh_SA_HLApI_CD165spike_2_R1
		PR		HLA-Ip	1.00E+08	1	B12	1/3	20170303_QEh1_LC2_FaMa_ChCh_SA_HLApI_CD165spike_3_R1
CM467	BR	PR		HLA-Ip	1.00E+08	1	A11	1/3	20170303_QEh1_LC2_FaMa_ChCh_SA_HLApI_CM647_1_R1
		PR		HLA-Ip	1.00E+08	1	A12	1/3	20170303_QEh1_LC2_FaMa_ChCh_SA_HLApI_CM647_2_R1
		PR		HLA-Ip	1.00E+08	1	B1	1/3	20170303_QEh1_LC2_FaMa_ChCh_SA_HLApI_CM647_3_R1
JY	BR	PR		HLA-Ip	1.00E+08	1	A9	1/3	20170303_QEh1_LC2_FaMa_ChCh_SA_HLApI_JY_1_R1_exp2
		PR		HLA-Ip	1.00E+08	1	A10	1/3	20170303_QEh1_LC2_FaMa_ChCh_SA_HLApI_JY_2_R1_exp2
		PR		HLA-Ip	1.00E+08	1	B10	1/3	20170303_QEh1_LC2_FaMa_ChCh_SA_HLApI_JY_3_R1_exp2
PD42	BR	PR		HLA-Ip	1.00E+08	1	B3	1/3	20170303_QEh1_LC2_FaMa_ChCh_SA_HLApI_PD42_1_R1
		PR		HLA-Ip	1.00E+08	1	B4	1/3	20170303_QEh1_LC2_FaMa_ChCh_SA_HLApI_PD42_2_R1
		PR		HLA-Ip	1.00E+08	1	B5	1/3	20170303_QEh1_LC2_FaMa_ChCh_SA_HLApI_PD42_3_R1
TIL1	BR	PR		HLA-Ip	1.00E+08	1	A5	1/3	20170303_QEh1_LC2_FaMa_ChCh_SA_HLApI_TIL1_1_R1
		PR		HLA-Ip	1.00E+08	1	A6	1/3	20170303_QEh1_LC2_FaMa_ChCh_SA_HLApI_TIL1_2_R1
		PR		HLA-Ip	1.00E+08	1	A7	1/3	20170303_QEh1_LC2_FaMa_ChCh_SA_HLApI_TIL1_3_R1
TIL3	BR	PR		HLA-Ip	1.00E+08	1	B7	1/3	20170306_QEh1_LC2_FaMa_ChCh_SA_HLApI_TIL3_1_R1
		PR		HLA-Ip	1.00E+08	1	B8	1/3	20170306_QEh1_LC2_FaMa_ChCh_SA_HLApI_TIL3_2_R1
		PR		HLA-Ip	1.00E+08	1	B9	1/3	20170306_QEh1_LC2_FaMa_ChCh_SA_HLApI_TIL3_3_R1
CD165	BR	PR		HLA-IIp	1.00E+08	1	A4	1/3	20170317_QEh1_LC2_FaMa_ChCh_SA_HLApII_CD165spike_1_R1
		PR		HLA-IIp	1.00E+08	1	B2	1/3	20170317_QEh1_LC2_FaMa_ChCh_SA_HLApII_CD165spike_2_R1
		PR		HLA-IIp	1.00E+08	1	B12	1/3	20170317_QEh1_LC2_FaMa_ChCh_SA_HLApII_CD165spike_3_R2
CM467	BR	PR		HLA-IIp	1.00E+08	1	A11	1/3	20170317_QEh1_LC2_FaMa_ChCh_SA_HLApII_CM647_1_R1
		PR		HLA-IIp	1.00E+08	1	A12	1/3	20170317_QEh1_LC2_FaMa_ChCh_SA_HLApII_CM647_2_R1
		PR		HLA-IIp	1.00E+08	1	B1	1/3	20170317_QEh1_LC2_FaMa_ChCh_SA_HLApII_CM647_3_R1
PD42	BR	PR		HLA-IIp	1.00E+08	1	B3	1/3	20170317_QEh1_LC2_FaMa_ChCh_SA_HLApII_PD42_1_R1
		PR		HLA-IIp	1.00E+08	1	B4	1/3	20170317_QEh1_LC2_FaMa_ChCh_SA_HLApII_PD42_2_R1
		PR		HLA-IIp	1.00E+08	1	B5	1/3	20170317_QEh1_LC2_FaMa_ChCh_SA_HLApII_PD42_3_R1
		PR		HLA-IIp	1.00E+08	1	A1	1/3	20170317_QEh1_LC2_FaMa_ChCh_SA_HLApII_RA957_1_R1

RA957	BR	PR	HLA-IIp	1.00E+08	1	A2	1/3	20170317_QEh1_LC2_FaMa_ChCh_SA_HLApII_RA957_2_R1
		PR	HLA-IIp	1.00E+08	1	A3	1/3	20170317_QEh1_LC2_FaMa_ChCh_SA_HLApII_RA957_3_R1
TIL1	BR	PR	HLA-IIp	1.00E+08	1	A5	1/3	20170317_QEh1_LC2_FaMa_ChCh_SA_HLApII_TIL1_1_R1
		PR	HLA-IIp	1.00E+08	1	A6	1/3	20170317_QEh1_LC2_FaMa_ChCh_SA_HLApII_TIL1_2_R1
		PR	HLA-IIp	1.00E+08	1	A7	1/3	20170303_QEh1_LC2_FaMa_ChCh_SA_HLApII_TIL1_3_R1
TIL3	BR	PR	HLA-IIp	1.00E+08	1	B7	1/3	20170317_QEh1_LC2_FaMa_ChCh_SA_HLApII_TIL3_1_R1
		PR	HLA-IIp	1.00E+08	1	B8	1/3	20170317_QEh1_LC2_FaMa_ChCh_SA_HLApII_TIL3_2_R1
		PR	HLA-IIp	1.00E+08	1	B9	1/3	20170317_QEh1_LC2_FaMa_ChCh_SA_HLApII_TIL3_3_R1
JY	BR	PR	HLA-IIp	1.00E+08	1	A9	1/3	20170320_QEh1_LC2_FaMa_ChCh_SA_HLApII_JY_1_R1_exp2_R1
		PR	HLA-IIp	1.00E+08	1	A10	1/3	20170320_QEh1_LC2_FaMa_ChCh_SA_HLApII_JY_1_R2_exp2_R1
		PR	HLA-IIp	1.00E+08	1	B10	1/3	20170320_QEh1_LC2_FaMa_ChCh_SA_HLApII_JY_2_R1_exp2_R1
mock					1	A8		
					1	B6		
					1	B11		
3830-NJF	BR		HLA-Ip	0.7-1 g	2	C6	1/3	20170320_QEh1_LC2_FaMaChCh_SA_HLApI_3830-NJF_1_R1
	BR		HLA-Ip	0.7-1 g	2	C7	1/3	20170320_QEh1_LC2_FaMaChCh_SA_HLApI_3830-NJF_2_R1
	BR		HLA-Ip	0.7-1 g	2	C8	1/3	20170320_QEh1_LC2_FaMaChCh_SA_HLApI_3830-NJF_3_R1
3849-BR	BR		HLA-Ip	0.7-1 g	2	C9	1/3	20170311_QEh1_LC2_FaMaChCh_SA_HLApI_3849-BR_2_R2
	BR		HLA-Ip	0.7-1 g	2	C10	1/3	20170311_QEh1_LC2_FaMaChCh_SA_HLApI_3849-BR_3_R1
3912-BAM	BR		HLA-Ip	1.47 g	2	C11	1/3	20170311_QEh1_LC2_FaMaChCh_SA_HLApI_3912-BAM_1_R1
3865-DM	BR		HLA-Ip	1.39 g	2	C12	1/3	201703011_QEh1_LC2_FaMaChCh_SA_HLApI_3865-DM_1_R1
3830-NJF	BR		HLA-IIp	0.7-1 g	2	C6	1/3	20170320_QEh1_LC2_FaMaChCh_SA_HLApII_3830-NJF_1_R1
	BR		HLA-IIp	0.7-1 g	2	C7	1/3	20170320_QEh1_LC2_FaMaChCh_SA_HLApII_3830-NJF_2_R1
	BR		HLA-IIp	0.7-1 g	2	C8	1/3	20170320_QEh1_LC2_FaMaChCh_SA_HLApII_3830-NJF_3_R1
3849-BR	BR		HLA-IIp	0.7-1 g	2	C9	1/3	20170320_QEh1_LC2_FaMaChCh_SA_HLApII_3849-BR_2_R1
	BR		HLA-IIp	0.7-1 g	2	C10	1/3	20170320_QEh1_LC2_FaMaChCh_SA_HLApII_3849-BR_3_R1
3912-BAM	BR		HLA-IIp	1.47 g	2	C11	1/3	20170320_QEh1_LC2_FaMaChCh_SA_HLApII_3912-BAM_1_R1
3865-DM	BR		HLA-IIp	1.39 g	2	C12	1/3	201703020_QEh1_LC2_FaMaChCh_SA_HLApII_3865-DM_1_R1
	BR	PR	HLA-Ip	1.00E+07	3	D5	1/3	20170402_QEh1_LC1_FaMaChCh_SA_CD165_10M_1_R1
		PR	HLA-Ip	3.00E+07	3	D6	1/3	20170402_QEh1_LC1_FaMaChCh_SA_CD165_30M_1_R1
		PR	HLA-Ip	5.00E+07	3	D7	1/3	20170402_QEh1_LC1_FaMaChCh_SA_CD165_50M_1_R1
		PR	HLA-Ip	7.00E+07	3	D8	1/3	20170413_QEh1_LC2_ChC_FaMa_SA_HLApI_CD165_70Mnew_1_R1
		PR	HLA-Ip	1.00E+08	1	A4	1/3	20170303_QEh1_LC2_FaMa_ChCh_SA_HLApI_CD165spike_1_R1
	BR	PR	HLA-Ip	1.00E+07	3	E5	1/3	20170402_QEh1_LC1_FaMaChCh_SA_CD165_10M_2_R1
		PR	HLA-Ip	3.00E+07	3	E6	1/3	20170402_QEh1_LC1_FaMaChCh_SA_CD165_30M_2_R1
		PR	HLA-Ip	5.00E+07	3	E7	1/3	20170402_QEh1_LC1_FaMaChCh_SA_CD165_50M_2_R1
		PR	HLA-Ip	7.00E+07	3	E8	1/3	20170413_QEh1_LC2_ChC_FaMa_SA_HLApI_CD165_70Mnew_2_R1
		PR	HLA-Ip	1.00E+08	1	B2	1/3	20170303_QEh1_LC2_FaMa_ChCh_SA_HLApI_CD165spike_2_R1
		PR	HLA-Ip	1.00E+07	3	F5	1/3	20170402_QEh1_LC1_FaMaChCh_SA_CD165_10M_3_R1

		PR	HLA-Ip	3.00E+07	3	F6	1/3	20170402_QEh1_LC1_FaMaChCh_SA_CD165_30M_3_R1
	BR	PR	HLA-Ip	5.00E+07	3	F7	1/3	20170402_QEh1_LC1_FaMaChCh_SA_CD165_50M_3_R1
		PR	HLA-Ip	7.00E+07	3	F8	1/3	20170413_QEh1_LC2_ChC_FaMa_SA_HLApI_CD165_70Mnew_3_R1
CD165		PR	HLA-Ip	1.00E+08	1	B12	1/3	20170303_QEh1_LC2_FaMa_ChCh_SA_HLApI_CD165spike_3_R1
		PR	HLA-IIp	1.00E+07	3	D5	1/3	20170403_QEh1_LC1_FaMaChCh_SA_HLApII_CD165_10M_1_R1
		PR	HLA-IIp	3.00E+07	3	D6	1/3	20170403_QEh1_LC1_FaMaChCh_SA_HLApII_CD165_30M_1_R1
	BR	PR	HLA-IIp	5.00E+07	3	D7	1/3	20170413_QEh1_LC2_ChC_FaMa_SA_HLApII_CD165_50Mmore_1_R1
		PR	HLA-IIp	7.00E+07	3	D8	1/3	20170405_QEh1_LC1_FaMaChCh_SA_HLApII_CD165_70M_1_R1
		PR	HLA-IIp	1.00E+08	1	A4	1/3	20170317_QEh1_LC2_FaMa_ChCh_SA_HLApII_CD165spike_1_R1
		PR	HLA-IIp	1.00E+07	3	E5	1/3	20170403_QEh1_LC1_FaMaChCh_SA_HLApII_CD165_10M_2_R1_170404100220
		PR	HLA-IIp	3.00E+07	3	E6	1/3	20170403_QEh1_LC1_FaMaChCh_SA_HLApII_CD165_30M_2_R1
	BR	PR	HLA-IIp	5.00E+07	3	E7	1/3	20170413_QEh1_LC2_ChC_FaMa_SA_HLApII_CD165_50Mmore_2_R1
		PR	HLA-IIp	7.00E+07	3	E8	1/3	20170405_QEh1_LC1_FaMaChCh_SA_HLApII_CD165_70M_2_R1
		PR	HLA-IIp	1.00E+08	1	B2	1/3	20170317_QEh1_LC2_FaMa_ChCh_SA_HLApII_CD165spike_2_R1
		PR	HLA-IIp	1.00E+07	3	F5	1/3	20170403_QEh1_LC1_FaMaChCh_SA_HLApII_CD165_10M_3_R1
		PR	HLA-IIp	3.00E+07	3	F6	1/3	20170403_QEh1_LC1_FaMaChCh_SA_HLApII_CD165_30M_3_R1
	BR	PR	HLA-IIp	5.00E+07	3	F7	1/3	20170413_QEh1_LC2_ChC_FaMa_SA_HLApII_CD165_50Mmore_3_R1
		PR	HLA-IIp	7.00E+07	3	F8	1/3	20170405_QEh1_LC1_FaMaChCh_SA_HLApII_CD165_70M_3_R1
		PR	HLA-IIp	1.00E+08	1	B12	1/3	20170317_QEh1_LC2_FaMa_ChCh_SA_HLApII_CD165spike_3_R2
	BR		HLA-Ip	1.50E+08	4	G1	1/2	20170608_QEh1_LC1_ChCh_FAMA_SA_HLAIp_UWB1289_ctrl_1_R2
UWB.1 289 ctrl	BR		HLA-Ip	1.50E+08	4	G2	1/2	20170608_QEh1_LC1_ChCh_FAMA_SA_HLAIp_UWB1289_ctrl_2_R2
	BR		HLA-Ip	1.50E+08	4	G3	1/2	20170608_QEh1_LC1_ChCh_FAMA_SA_HLAIp_UWB1289_ctrl_3_R2
	BR		HLA-Ip	1.50E+08	4	G4	1/2	20170608_QEh1_LC1_ChCh_FAMA_SA_HLAIp_UWB1289_ctrl_4_R2
	BR		HLA-Ip	1.50E+08	4	G5	1/2	20170608_QEh1_LC1_ChCh_FAMA_SA_HLAIp_UWB1289_IFNg_1_R2
UWB.1 289 IFNg	BR		HLA-Ip	1.50E+08	4	G6	1/2	20170608_QEh1_LC1_ChCh_FAMA_SA_HLAIp_UWB1289_IFNg_2_R2
	BR		HLA-Ip	1.50E+08	4	G7	1/2	20170608_QEh1_LC1_ChCh_FAMA_SA_HLAIp_UWB1289_IFNg_3_R2
	BR		HLA-Ip	1.50E+08	4	G8	1/2	20170608_QEh1_LC1_ChCh_FAMA_SA_HLAIp_UWB1289_IFNg_4_R2
	BR		HLA-Ip	1.00E+08			1/3	20170609_QEh1_LC1_ChCh_FAMA_SA_HLAIp_JY_090517_3_R1
	BR		HLA-Ip	1.00E+08			1/3	20170609_QEh1_LC1_ChCh_FAMA_SA_HLAIp_JY_160517_1_R1
JY	BR		HLA-Ip	1.00E+08	different plates		1/3	20170609_QEh1_LC1_ChCh_FAMA_SA_HLAIp_JY_210417_2_R1
	BR		HLA-IIp	1.00E+08			1/3	20170609_QEh1_LC1_ChCh_FAMA_SA_HLAIIp_JY_090517_3_R1
	BR		HLA-IIp	1.00E+08			1/3	20170609_QEh1_LC1_ChCh_FAMA_SA_HLAIIp_JY_160517_1_R1
	BR		HLA-IIp	1.00E+08			1/3	20170609_QEh1_LC1_ChCh_FAMA_SA_HLAIIp_JY_210417_2_R1

BR: Separate cells pellets/tissue sections are biological replicates
PR: BR loaded onto several wells are plate replicates
MSR: PR measured twice are MS replicates
HLA-Ip: HLA class I peptides
HLA-IIp: HLA class II peptides
