# Supplementary material for: High-throughput and Sensitive Immunopeptidomics Platform Reveals Profound Interferonγ-Mediated Remodeling of the Human Leukocyte Antigen (HLA) Ligandome
Source: Mol Cell Proteomics. 2017 Dec 14;17(3):533–48. doi: 10.1074/mcp.TIR117.000383 (PMC5836376; doi:10.1074/mcp.TIR117.000383)
Supplement: Supplemental Data [file supp_TIR117.000383_132937_2_supp_37763_p0v94k.pdf]

Table S3. Detailed MS/MS information about the 15 isotopically heavy labeled synthetic peptides used as spiked-in standards. The retention times were extracted from synthetic and their endogenous counterparts in the CD 165 HLA-Ip samples.

		Heavy			light			
Mass shift	Sequence	Th. Mass 1+	Th. Mass 2+	Th. Mass 3+	Th. Mass 1+	Th. Mass 2+	Th. Mass 3+	RT
7.0172	RYKEENNDH(L)	1324.6354	662.8213	442.2166	1317.6182	659.3127	439.8776	17.43
4.0071	TEYDSRVK(A)	1072.53909	536.773495	358.1849633	1068.532	534.7696	356.8489	22.59
7.0172	KQAEIVKR(L)	1091.7009	546.3541	364.5718	1084.6837	542.8455	362.2328	26.32
**7.0172**	**IYTSSVNR(L)**	**1059.5906**	**530.299**	**353.8684**	**1052.5735**	**526.7904**	**351.5293**	**42.05**
4.0071	SEVTFALHS(A)	1065.53329	533.270595	355.8496967	1061.5262	531.2667	354.5136	52.26
4.0071	QEVDRIKE(A)	1091.58129	546.294595	364.5323633	1087.5742	544.2907	363.1963	23.92
4.0071	LEKQDWEH(A)	1159.54999	580.278945	387.1885967	1155.5429	578.2751	385.8525	30.21
**7.0172**	**KVKDDVDK(L)**	**1066.6216**	**533.8144**	**356.2121**	**1059.6045**	**530.3059**	**353.873**	**24.09**
4.0071	REYDGNIRQ(A)	1225.60419	613.306045	409.2066633	1221.5971	611.3022	407.8705	23.42
4.0071	AERDFIHT(A)	1063.52889	532.268395	355.1815633	1059.5218	530.2645	353.8454	33.54
4.0071	KEWDEFQK(A)	1184.57039	592.789145	395.52873	1180.5633	590.7853	394.1926	38.68
**4.0071**	**QVISQAAVVH(A)**	**1126.63369**	**563.820795**	**376.2164967**	**1122.6266**	**561.8169**	**374.8804**	**43.95**
4.0071	AESYYRHA(A)	1071.49759	536.252745	357.8377967	1067.4905	534.2489	356.5017	20
7.0172	SQAPAVNT(L)	907.4957	454.2515	303.1701	900.4785	450.7429	300.831	46.33
7.0172	KLKGKEAE(L)	1022.6318	511.8195	341.5488	1015.6146	508.3109	339.2097	17.65
