# Supplementary material for: High-throughput and Sensitive Immunopeptidomics Platform Reveals Profound Interferonγ-Mediated Remodeling of the Human Leukocyte Antigen (HLA) Ligandome
Source: Mol Cell Proteomics. 2017 Dec 14;17(3):533–48. doi: 10.1074/mcp.TIR117.000383 (PMC5836376; doi:10.1074/mcp.TIR117.000383)
Supplement: Supplemental Data [file supp_TIR117.000383_132937_2_supp_37771_p0v9hm.pdf]

Table S4. Detailed MS/MS information about 3 selected isotopically heavy labeled synthetic peptides and their light counterparts used to measure technical reproducibility between the three replicates. Area under the curve, AUC; standard deviation, SD; coefficient variation, CV.

Statistic on 3 peptides									
		Heavy AUC		Light AUC		Ratio H/L			
Sequence	Sample	sum intensities of all charge states (1+,2+,3+)	log2(sum)	sum intensities of all charge states (1+,2+,3+)	log2(sum)	log2(H)/log2(L)	mean	SD	CV
KVKDDVDK(L)	CD165-HLA-I-R1	1.0644E+10	33.31	2.3139E+08	27.79	1.20	1.22	0.01	0.01
	CD165-HLA-I-R2	1.2504E+10	33.54	2.0281E+08	27.60	1.22			
	CD165-HLA-I-R3	1.6221E+10	33.92	1.7123E+08	27.35	1.24			
IYTSSVNR(L)	CD165-HLA-I-R1	7.0302E+09	32.71	2.6195E+09	31.29	1.05	1.06	0.01	0.01
	CD165-HLA-I-R2	1.1108E+10	33.37	3.1283E+09	31.54	1.06			
	CD165-HLA-I-R3	1.8435E+10	34.10	3.0362E+09	31.50	1.08			
QVISQAAVVH(A)	CD165-HLA-I-R1	3.1127E+09	31.54	9.8878E+08	29.88	1.06	1.07	0.01	0.01
	CD165-HLA-I-R2	4.6103E+09	32.10	1.1297E+09	30.07	1.07			
	CD165-HLA-I-R3	7.9096E+09	32.88	1.1323E+09	30.08	1.09			
