# Supplementary material for: High-throughput and Sensitive Immunopeptidomics Platform Reveals Profound Interferonγ-Mediated Remodeling of the Human Leukocyte Antigen (HLA) Ligandome
Source: Mol Cell Proteomics. 2017 Dec 14;17(3):533–48. doi: 10.1074/mcp.TIR117.000383 (PMC5836376; doi:10.1074/mcp.TIR117.000383)
Supplement: Supplemental Data [file supp_TIR117.000383_132937_2_supp_37776_p0v9xx.pdf]

Table S5. Literature on HLA-I and HLA-II immunopeptidomics.

	Authors/Journal		Sample preparation									MS measurement		Informatics			
	Authors	Journal, year	Sample type	Crosslinker	Antibody to bead Ratio	Number of cells per mL lysis buffer	Number of cells	Number of cells/antibody-linked beads	IP conditions	HLAIp Elution	Separation of peptides	MS	MS/MS type	Software Analysis	FDR applied	Total unique peptides	Raw files deposited
HLA class I studies	Ritz D. et al	Proteomics, 2016	6 cell lines	n.a	n.a	5 x 10^7/mL	1 x 10^8	n.a	2h at 4°C	0.1 M acetic acid	MWCO (10 kDA) and C18-resin (for cell lines, only C18)	Q Exactive MS	HCD	SEQUEST, MaxQuant	n.a, 1%	4000-7000	n.a
	Ternette N. et al	European Journal of Immunology,2016	4 cell lines/types	n.a	5 mg/mL	10 mL for 10^9	1 x 10^8	n.a	n.a	5 mL 10% Acetic acid	off-line C18 fractionation	Triple-TOF 5600, Orbitrap Elite	CID, CID	PEAKS, MASCOT	5%	2416-6795	n.a
	Pymm P. et al	Nature Structural and Molecular Biology, 2017	2 cell types	DMP	10 mg/mL	n.a	5 x 10^9	2 mL beads	n.a	10% acetic acid	off-line C18 fractionation	Triple-TOF 5600plus	CID	ProteinPilot	5%	8233, 8432 (non-transfected, transfected)	n.a
	Bassani-Sternberg M. et al	Molecular and Cellular Proteomics, 2015	7 cell lines	n.a	n.a	n.a	5 x 10^8	n.a	n.a	500 µL 0.1 N acetic acid, 7 elutions	C18 (twice)	Q Exactive MS	HCD	MaxQuant	1%	2500-5500	yes
	Abelin JG. et al	Immunity, 2017	16 cell lines	no crosslinker	n.a	2.5- 5 x 10^7/mL	5-10 x 10^7	20 µL	3h	3% ACN/5% FA, 1% formic acid and 10% acetic acid	in-house C18 stage tip	Q Exactive HF and Q ExactivePlus	HCD	Spectrum Mill	1-2%	1000-3500	yes
	Khodadoust MS. et al	Nature, 2017	2 cell lines	n.a	n.a	n.a	1x 10^8-10^9	n.a	5h at 4°C	10% acetic acid	10kDa molecular weight cut-off size filter and C18 based Stage tips	LTQ Orbitrap Elite	HCD or CID	SEQUEST and PEAKS DB	1%	8000-13000	n.a
	Zarling AL. et al	PNAS, 2006	4 cell lines	n.a	n.a	n.a	2-5 x 10^9	n.a	n.a	0.2 M acetic acid	filtration through 5 kDa cutoff filter	FTICR	CID	MASCOT	n.a	>10000 peptides	n.a
	Hassan C. et al	Molecular and Cellular Proteomics, 2013	2 cell lines	DMP	2.5 mg/mL	0.1 x 10^9 cells /mL	4-6 x 10^{10}	n.a	2.5 mL/min	10% acetic acid	10 kDa membrane (Pall macrosep centrifuge devices),SCF, IEF, and RP C18	LTQ-FT Ultra MS	CID	MASCOT	n.a	HHC: 11000 peptides JY: 6500	yes
	Walz S. et al	Blood, 2015	6 cell lines	CNBr	1mg/40 mg	1 mL pellet / 3 mL lysis buffer	1 mL cell pellet	1 mL pellet/1 mL beads	o.n	0.2% TFA (40 µL per 40 mg of stationary phase)	ultrafiltration through 10 kDa cut off membrane and C18 Zip Tips	LTQ Orbitrap XL	CID	MASCOT	5%	1000-4000	n.a
	Alpizar A. et al	Molecular and Cellular Proteomics, 2017	1 cell line	n.a	n.a	n.a	1 x 10^{10}	n.a	n.a	0.1% TFA	Vivacon 2 and OMIX C18 tip	Orbitrap Elite	EThcD	MASCOT, X!tandem2, MyriMatch, OMSSA	1% (combined)	HLA B40: 7375	yes
	Di Marco C. et al	Journal of Immunology, 2017	17 cell lines	CNBr	1mg/40 mg	1 mL pellet / 3 mL lysis buffer	2.5 x 10^9	1 mL pellet/1 mL beads	o.n	0.2% TFA	ultrafiltration through 10 kDa cut off membrane and C18 Zip Tips	Orbitrap Fusion Lumos	CID	SEQUEST Proteome Discoverer	5%	632-3463	n.a

HLA class II studies	Walz S. et al	Blood, 2015	6 cell lines	CNBr	1mg/40 mg	1 mL pellet / 3 mL lysis buffer	1 mL cell pellet	1 mL pellet/ 1 mL beads	o.n	0.2% TFA (40 µL per 40 mg of stationary phase)	ultrafiltration through 10 kDa cut off membrane and C18 Zip Tips	LTQ Orbitrap XL	CID	MASCOT	5%	200-1800	n.a
	Khodadoust MS. et al	Nature, 2017	2 cell lines	n.a	n.a	n.a	1x 10^8-10^9	n.a	5h at 4°C	10% acetic acid	10kDa molecular weight cut-off size filter and C18 based Stage tips	LTQ Orbitrap Elite	HCD or CID	SEQUEST and PEAKS DB	1%	7000-9000	n.a
	Heyder T. et al	Molecular and Cellular Proteomics, 2016	PRIESS and bronchoalveolar lavage (BAL) cells from 7 donors	none	1 to 1	800 µL lysis buffer	1 x 10^7 (B cell line), 1 -5 x 10 ^{7}(BAL)	1 x 10^7 cells / 40 uL beads	2h at RT	50% acetonitrile, 5% formic acid (5 bed volumes)	SCX	Qexactive, Qexactive Plus, LTQ Orbitrap Velos	HCD, HCD, CID	MASCOT	5%	0-250 (EBV-B-cell line PRIESS), 48-753 (BAL), 1500 peptides (total)	yes
	Ciudad M. T. et al	Journal of Leukocyte Biology, 2017	MoDCs from 7 patients	CNBr	5 mg/mL	n.a	30-66 x 10^6	n.a	o.n at 4°C	10% acetic acid, 15 min at 70°C	C18 StageTip	LTQ Orbitrap XL	CID	SEQUEST	1%	1319 peptides (total from 7 donors) 85-362 (unique peptides)	n.a
	van Haren S.D. et al	Molecular and Cellular Proteomics, 2011	MoDCs from 4 donors	CNBr	n.a	n.a	5 x 10^6	n.a	o. n at 4°C	10% acetic acid, 15 min at 70°C	C18 ZipTip	LTQ Orbitrap XL	CID	SEQUEST	n.a	270-930 peptides	n.a
	Adamopoulou E. et al	Nature Communications, 2013	Thymic DCs from 8 donors	CnBr	n.a	n.a	3 x 10^7	n.a	o.n at 4 °C	0.1% TFA	MW cutoff filter of 10 kDa	Waters Q-Tof Premier	CID	MassLynx 4.0	n.a	total: 221 peptides	n.a
	Klatt M.G. et al	Oncoimmunology, 2016	PBMCs and BMNCs from 30 donors	CnBr	1mg/40 mg	n.a	n.a	1 mL pellet/ 1 mL beads	o.n	0.2% TFA (40 µL per 40 mg of stationary phase)	ultrafiltration through 10 kDa cut off membrane and C18 Zip Tips	LTQ Orbitrap XL hybrid	CID	MASCOT	5%	4-784 identified peptides	n.a
	Costantino C. et al	PlosONE, 2012	B- and T cell lines from 2 donors	CnBr	n.a	n.a	10^7-10^8	n.a	n.a	0.1% TFA	n.a	LTQ	CID	SEQUEST	n.a	30-213 peptides	n.a
